# *De novo* sequencing and analysis of the *Ulva linza* transcriptome to discover putative mechanisms associated with its successful colonization of coastal ecosystems

**DOI:** 10.1186/1471-2164-13-565

**Published:** 2012-10-25

**Authors:** Xiaowen Zhang, Naihao Ye, Chengwei Liang, Shanli Mou, Xiao Fan, Jianfang Xu, Dong Xu, Zhimeng Zhuang

**Affiliations:** 1 Yellow Sea Fisheries Research Institute, Chinese Academy of Fishery Sciences, Qingdao, 266071, China; 2 Qingdao University of Science > Technology, Qingdao, 266042, China; 3 Key Laboratory of Marine Bioactive Substance, The First Institute of Oceanography, State Oceanic administration (SOA), Qingdao, 266061, China

## Abstract

**Background:**

The green algal genus Ulva Linnaeus (Ulvaceae, Ulvales, Chlorophyta) is well known for its wide distribution in marine, freshwater, and brackish environments throughout the world. The *Ulva* species are also highly tolerant of variations in salinity, temperature, and irradiance and are the main cause of green tides, which can have deleterious ecological effects. However, limited genomic information is currently available in this non-model and ecologically important species. *Ulva linza* is a species that inhabits bedrock in the mid to low intertidal zone, and it is a major contributor to biofouling. Here, we presented the global characterization of the *U. linza* transcriptome using the Roche GS FLX Titanium platform, with the aim of uncovering the genomic mechanisms underlying rapid and successful colonization of the coastal ecosystems.

**Results:**

*De novo* assembly of 382,884 reads generated 13,426 contigs with an average length of 1,000 bases. Contiguous sequences were further assembled into 10,784 isotigs with an average length of 1,515 bases. A total of 304,101 reads were nominally identified by BLAST; 4,368 isotigs were functionally annotated with 13,550 GO terms, and 2,404 isotigs having enzyme commission (EC) numbers were assigned to 262 KEGG pathways. When compared with four other full sequenced green algae, 3,457 unique isotigs were found in *U. linza* and 18 conserved in land plants. In addition, a specific photoprotective mechanism based on both *Lhc*SR and *Psb*S proteins and a C4-like carbon-concentrating mechanism were found, which may help *U. linza* survive stress conditions. At least 19 transporters for essential inorganic nutrients (i.e., nitrogen, phosphorus, and sulphur) were responsible for its ability to take up inorganic nutrients, and at least 25 eukaryotic cytochrome P450s, which is a higher number than that found in other algae, may be related to their strong allelopathy. Multi-origination of the stress related proteins, such as glutamate dehydrogenase, superoxide dismutases, ascorbate peroxidase, catalase and heat-shock proteins, may also contribute to colonization of *U. linza* under stress conditions.

**Conclusions:**

The transcriptome of *U. linza* uncovers some potential genomic mechanisms that might explain its ability to rapidly and successfully colonize coastal ecosystems, including the land-specific genes; special photoprotective mechanism based on both *Lhc*SR and *Psb*S; development of C4-like carbon-concentrating mechanisms; muti-origin transporters for essential inorganic nutrients; multiple and complex P450s; and glutamate dehydrogenase, superoxide dismutases, ascorbate peroxidase, catalase, and heat-shock proteins that are related to stress resistance.

## Background

Green algae originated as much as 1500 million years ago, evolving shortly after the endosymbiotic event that gave rise to early photosynthetic eukaryotes [1]. During the evolutionary history of Earth, green algae have become major players in global energy/biomass production and biogeochemical recycling [2]. The green algal genus *Ulva* Linnaeus (Ulvaceae, Ulvales, Chlorophyta), which includes the genus formerly known as *Enteromorpha*[3], is a cosmopolitan intertidal macroalga and includes more than 100 species [4]. It is well known for its distribution in all aquatic habitats including freshwater, brackish environments, marine and fully saline environments throughout the warm temperate and tropical regions of the world [5-7]. This group also possesses features that provide them with a competitive edge for rapid and successful colonization in eutrophic conditions, namely a copious production of reproductive spore bodies; the ability to rapidly take up and store high amounts of inorganic nitrogen; and a wide tolerance to adverse environmental conditions such as temperature, light intensity, salinity, and anoxia [8,9]. In addition, when these algae grow under eutrophic conditions, they have the competitive advantage of rapid and successful colonization, resulting in a “green tide” [8,10,11], which has the following deleterious ecological effects: the uncoupling of biogeochemical cycles in sediments from those in the water column [9], a negative effect on seagrass beds due to shading, disruption of feeding by wading birds [12], the development of a lethal environment due to oxygen deficiency [13] and a shift from a high diversity mixture to low-diversity assemblages of fast growing annuals [14]. Because of their ecological effects, green tides have drawn considerable attention from scientists and governments [8,9,12].

Whole genome or transcriptome sequencing could elucidate the genetic basis for this physiological characteristic or exploit this group’s unique genetic resources based on global gene expression [15-17]. Over the last few years, the application of newly developed high-throughput sequencing technologies has allowed hundreds of thousands of high-quality sequence reads to be produced *de novo* from whole genome or transcriptome templates, thus enabling immediate inroads to genetic studies of organisms for which little or no sequence data exist [18]. For non-model organisms, sequencing and assembling of genomes remains challenging and costly, even when considering high throughput sequencing technology [19]. As an alternative, sequencing transcriptomes is less complex and provides fast and cost-effective access to the gene expression profile of an organism [16,20]. Transcriptomes present a valuable resource for accelerating gene discovery by expanding gene families [21,22], improving genome annotation [23], elucidating phylogenetic relationships [24], facilitating breeding programs for both plants and animals by providing SSR and SNP markers [25,26], and allowing large-scale expression analysis [27,28] and rapid identification of transcripts involved in specific biological processes [29]. Recent studies have demonstrated the success of 454 *de novo* sequencing, assembly and analysis of transcriptomes in non-model organisms with no prior genomic resources [30-33]. The generation of such large-scale sequence data will enable functional analyses that were previously limited to model organisms and their rapid application in ecologically important taxa [34].

To date, genomic information about algae is still limited compared to land plants. Moreover, algae are generally far more diverse and evolutionarily divergent than land plants [35]. Currently, nearly complete genomic information is available publicly for only five green microalgae species: *Chlamydomonas reinhardtti* (Chlorophyceae) [36], *Chlorella variabilis* (Trebouxiophyceae) [37], *Ostreococcus tauri* (Prasinophyceae) [38], *Micromonas* (Prasinophyceae) [39] and *Volvox carteri* (Chlorophyceae) [40]. To date, there is little genomic data available for the Ulvophyceae, a group that is diverse and evolutionarily divergent from all of the currently sequenced genomes of green microalgae. For the Ulvophyceae group, EST resources have been developed using Sanger sequencing in three species, *Acetabularia acetabulum*[35], *Ulva linza*[41] and *Ulva prolifera*[42]*.* A comprehensive description of the full complement of genes as a transcriptome, however, is still unavailable. *Ulva linza* is one of the species found in the mid to low intertidal on bedrock, and it is also a major contributor to biofouling [41]. It must cope with more dramatic changes than land plants because of tidal changes in light intensity, temperature, salinity, and wave action and with the biotic stresses characteristic of dense coastal ecosystems. In this study, we sequenced the transcriptome of *U. linza* under a wide variety of environmental stress conditions with the aim of uncovering some of potential genomic mechanisms that might explain its ability to rapidly and successfully colonize the coastal ecosystems and form green tides.

## Results and Discussion

### 454 sequencing and de novo assembly of the transcriptome

**Figure 1:**
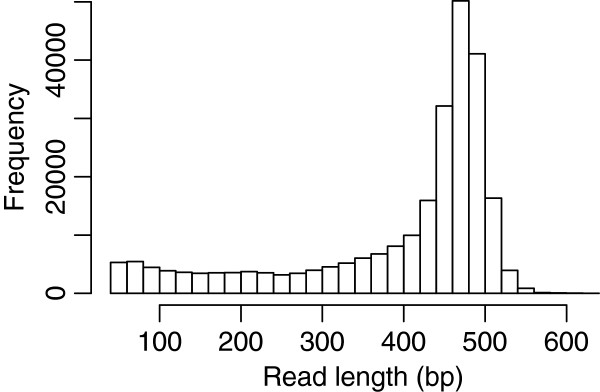
**Read length distribution of the *Ulva linza* transcriptome data.**

**Figure 2:**
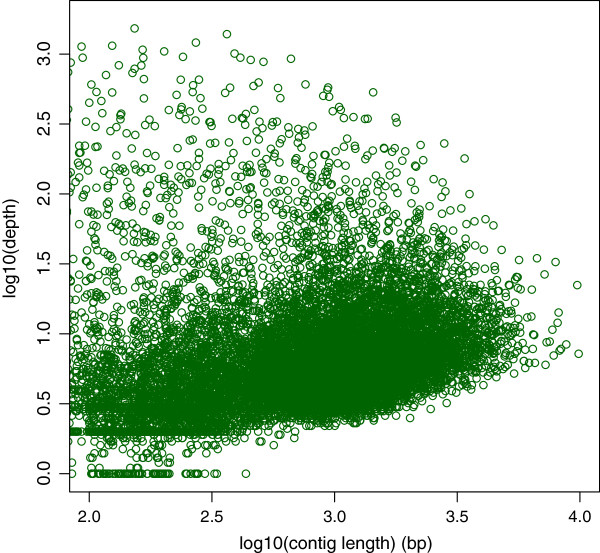
Contig length as a function of the contig read depth.

The normalized cDNA library of *U. linza* cells grown under the normal and six stress conditions was constructed using SMART technology. The library was subjected to a one-half plate run with the 454 GS FLX Titanium platform, and the transcriptome was assembled from the resulting sequencing reads. Sequencing of the cDNA library generated a total 503,789 raw reads (SRA053388), with an average sequence length of 396 bases (Figure 1, Table 1). After trimming the adapter sequences and removing the short sequences with low complexity and low quality scores, 445,787 high quality reads were obtained, corresponding to 88.5% of the original raw sequences. A total of 382,884 reads were assembled into 13,426 contiguous sequences (contigs), accounting for 85.9% of the assembled reads, while 38,961 reads remained as singletons (reads not assembled into contigs). The size of contigs ranged from 50 to 9,881 bases, with an average length of 1,000 bases. As expected for a randomly fragmented transcriptome, there was a positive relationship between the length of a given contig and the number of reads assembled into that contig. The length distribution and read depth of the contigs are shown in Figure 2, revealing that more than 9,578 contigs are greater than 400 bp, with an average depth of coverage of 14.48 sequences per nucleotide position. Contiguous sequences were further assembled into 10,784 isotigs, which are the putative transcripts constructed using the overlapping contig reads provided as input to the Newbler cDNA assembler. The average length of the isotigs was 1,515 bases, with an N50 of 1,856 bases (50% of the assembled bases were incorporated into isotigs greater than 1,856 bases). The coverage depth for isotigs ranged from 1 to 14, with an average of 1.24 contigs assembled into each isotig. When compared to the transcriptomes of green algae and land plants including *C. reinhardtii, V. carteri, P. patens* and *A. thaliana*, we found that a total of 6,519 (82.1%) common genes could be identified in the transcriptome of *U. linza*.

**Table 1:** Summary of the sequencing and assembly

	**Sequence (n)**	**Bases (bp)**
**Sequencing**		
Raw reads	503,789	199,554,589
Average read length	396 bp	
High-quality reads	445,787	175,500,089
Reads used in assembly	421,845	165,785,088
Average read length after trimming	393 bp	
**Contigs**		
Reads assembled as contigs	382,884	150,473,414
Number of contigs	13,426	13,438,442
Average length of contigs	1,000 bp	
Range of contig length	50-9,881 bp	
Depth on contigs	14.48	
**Isotigs**		
Number of isotigs	10,784	16,340,803
Average length of isotigs	1,515 bp	
Range of isotig length	50-13,194	
Depth on isotigs	1.24	

### Comparison to other proteomes of green algae

**Figure 3:**
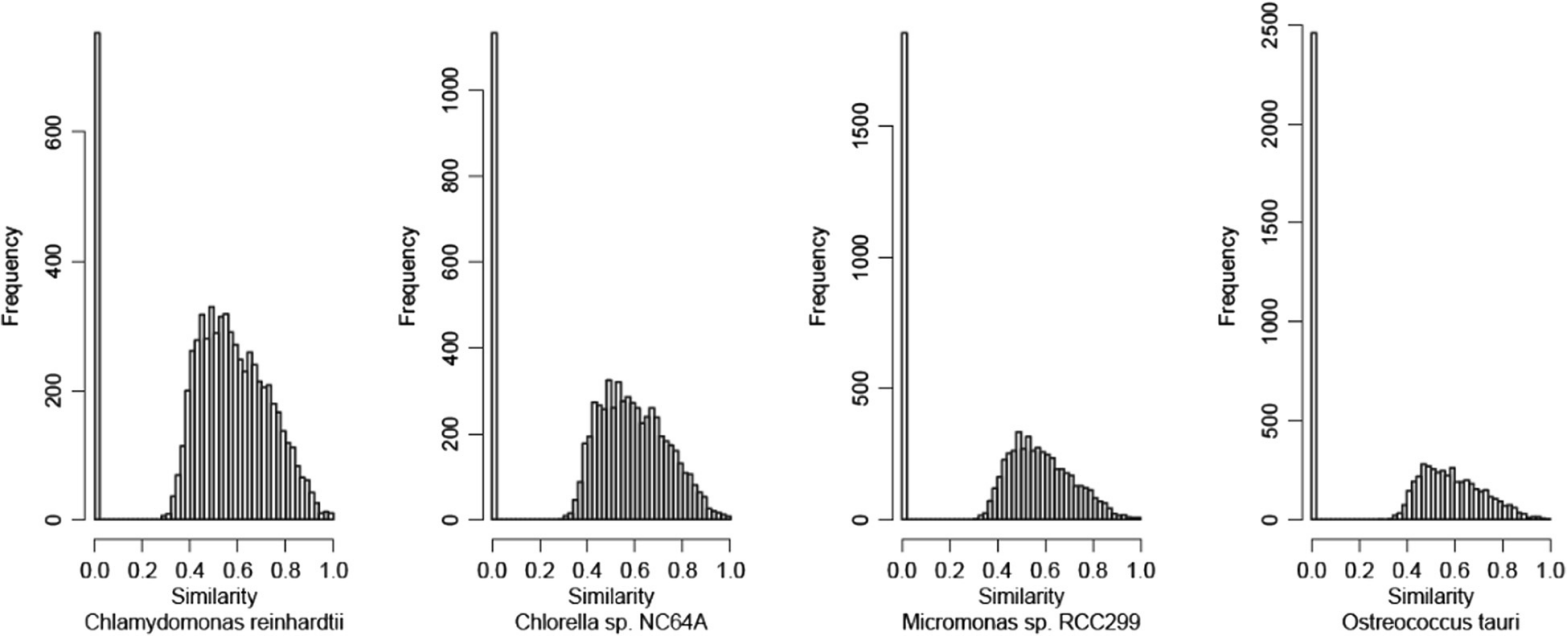
**Comparison between the *Ulva linza* transcriptome and four closely related chlorophyta genomes.**

To identify novel features of the *U. linza* transcriptome, a BLASTX search was used to compare the isotigs to sequences from other organisms belonging to the green algae lineage. In this analysis we included four fully sequenced organisms that diverged at different times during evolution: *C. reinhardtii*, a model organism for photosynthetic unicellular eukaryotes [36]; *O. tauri*, which is the smallest free-living eukaryote identified to date and one of the most ancient clade among Chlorophyta with the features of genome compaction and gene family downsizing [38]; *C. variabilis*, which is used as a model system for studying algal symbioses and algal–viral interactions of freshwater organisms [37]; and *Micromonas* which offers valuable insights into ecological differentiation and the dynamic nature of early plant evolution [39]. Sequence comparisons revealed that genes shared between *U. linza* and the four unicellular green microalgae have a consistent frequency and similarity (Figure 3). In contrast, the frequency of unique sequences when compared with different green algal species was clearly different, ranging from about 756 in *C. reinhardtii* to 2,485 in *O. tauri*. The 1,729 reads, which have no counterpart in *O. tauri* but do in *C. reinhardtii*, were assembled into 1,379 isotigs. Totally 762 isotigs could be annotated, among which at least 441 sequences were also conserved in *Volvox*, *Micromonas* and *Chlorella* (Additional file 1: Table S1). This indicates that *O. tauri* lost genes compared to other green algae.

**Figure 4:**
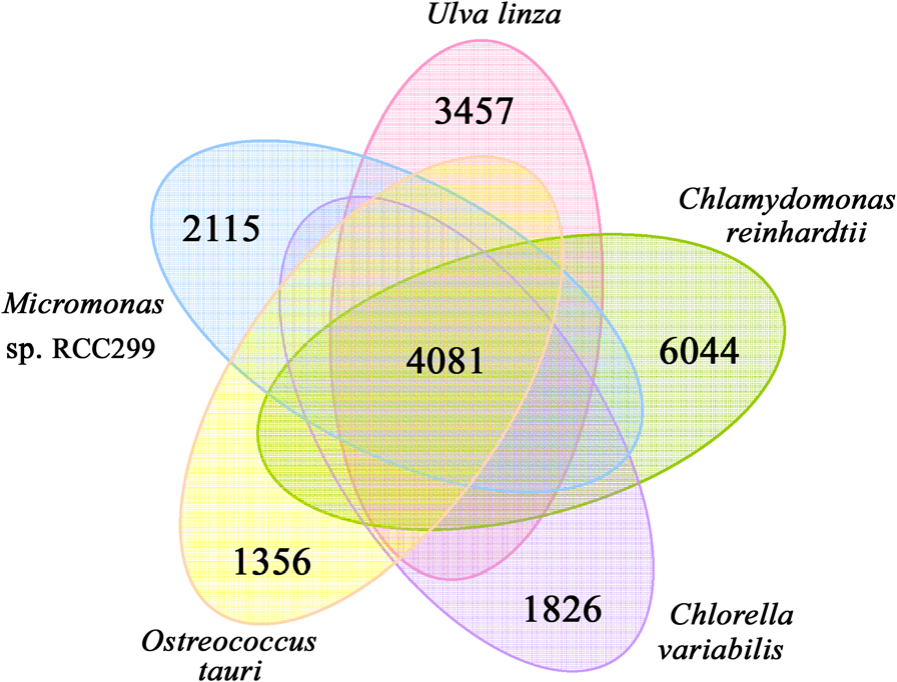
**Venn diagram representation of shared/unique genes of *U. linza* in comparison with four other sequenced green algae of *Chlamydomonas reinhardtii*, *Chlorella variabilis* NC64A, *Micromonas*sp. RCC299 and *Ostreococcus tauri.***

In total, 4,081 isotigs in *U. linza* were found to be conserved in all four of the green algae, thus they might have existed before the species diverged and may therefore play basic roles for their survival (Figure 4). The lineage-specific genes have evolved after the species diverged by gene transfer or gene loss, and may contribute to their specific characteristics and adaptation to their living environments. The analysis also provides us with 3,457 isotigs that are exclusive to *U. linza*, not found in the other four green algal genomes queried. This corresponds to 32.1% of the total isotig repertoire in *U. linza*, which is similar to the fraction of unique genes found in *Nannochloropis gaditana* (30.2%) [43] and *C. reinhardtii* (35.3%), but is much higher than that of *C. variabilis* NC64A (18.6%), *Micromonas* sp. RCC299 (21.0%) and *O. tauri* (17.5%). Among the 3,457 specific isotigs in *U. linza*, only 195 could be annotated and 18 of which were conserved in land plants including disease resistance proteins, an ABC transporter, etc. (Additional file 2: Table S2). *U. linza* lives in intertidal zone of coastal ecosystems, which is a transitional environment from sea water to land, and is therefore exposed to direct sunshine several hours per day. Moreover, the sessile form of life of *U. linza* cannot escape from stress conditions by swimming deeper, a behavior that is typical of planktonic algae [44]. We predicted that these land-specific genes in *U. linza* may be related to the colonization of the special coastal ecosystems.

### Functional annotation

All sequences were aligned against the local non-redundant (nr) protein database downloaded from the National Center for Biotechnology Information (NCBI) using the BLASTx algorithm. When the E-value cutoff was set at 10^-2^, a total of 304,101 reads (72.2% of total) had significant BLAST matches. According to the scores obtained by the BLASTX, the high quality reads were assigned to the taxonomic tree that was representative of Bacteria (10,685; 3.5%) and Eukaryota (218,725; 71.9%). As expected, the taxonomic assignment was well represented by Chlorophyta (90,074; 29.6%) and Embryophyta (11,229; 3.7%) within the Viridiplantae group. Within Chlorophyta, the top four assignments were assigned to *Chlorella*, *Ulva*, *Volvox* and *Chlamydomonas*.

**Figure 5:**
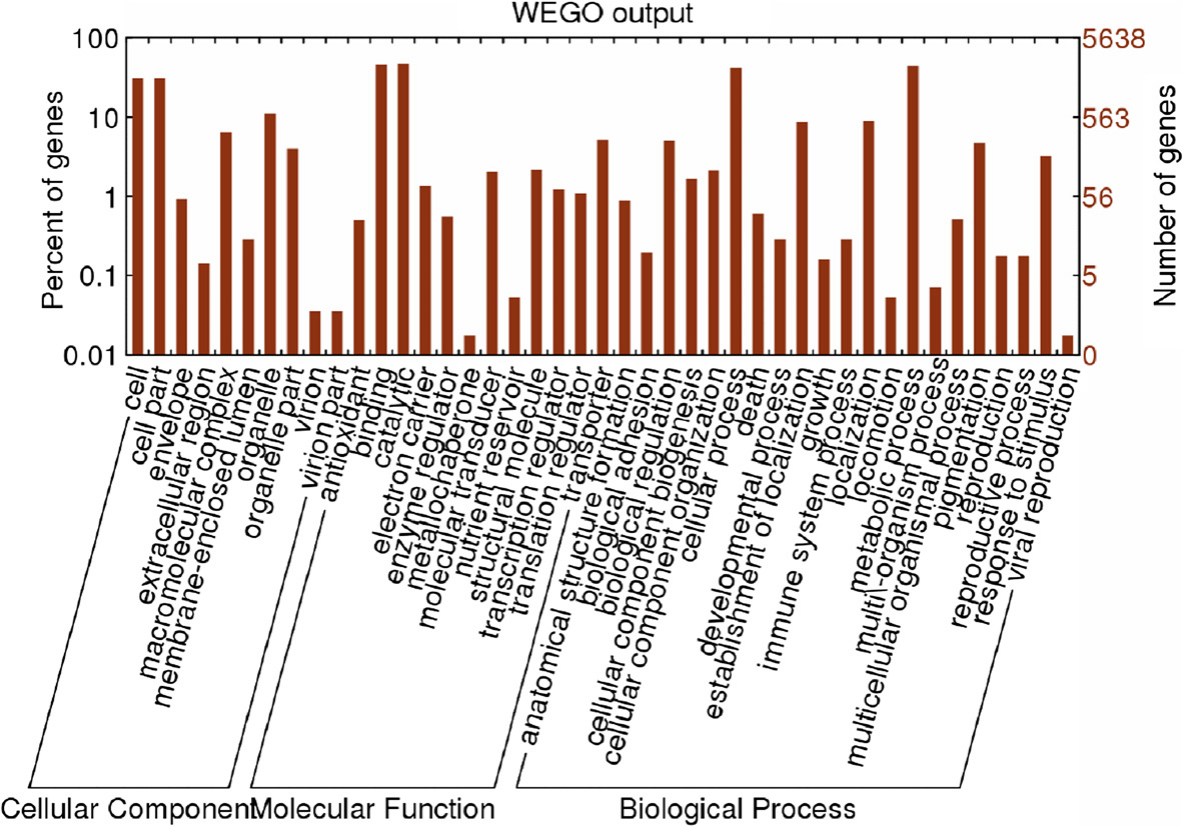
Functional categories based on GO annotations.

The isotigs were further annotated with GO terms based on their sequence similarities to known proteins in the UniProt database annotated with GO terms as well as the InterPro and Pfam domains they contain. A total of 4,368 isotigs (40.5%) were functionally annotated with 13,550 GO terms. Among these, 7,192 were assigned at least one GO term in the biological process category, 6,108 in the molecular function category and 3,233 in the cellular component category (Figure 5). In the biological process class (second level GO terms), the majority of the GO terms were grouped into cellular (42.7%) and metabolic (45.2%) processes. However, in the category of molecular function, the vast majority of GO terms were classified into binding (46.7%) and catalytic activities (47.4%). Under the category of cellular components, 32% of all GO terms corresponded to cell parts and organelles.

**Figure 6:**
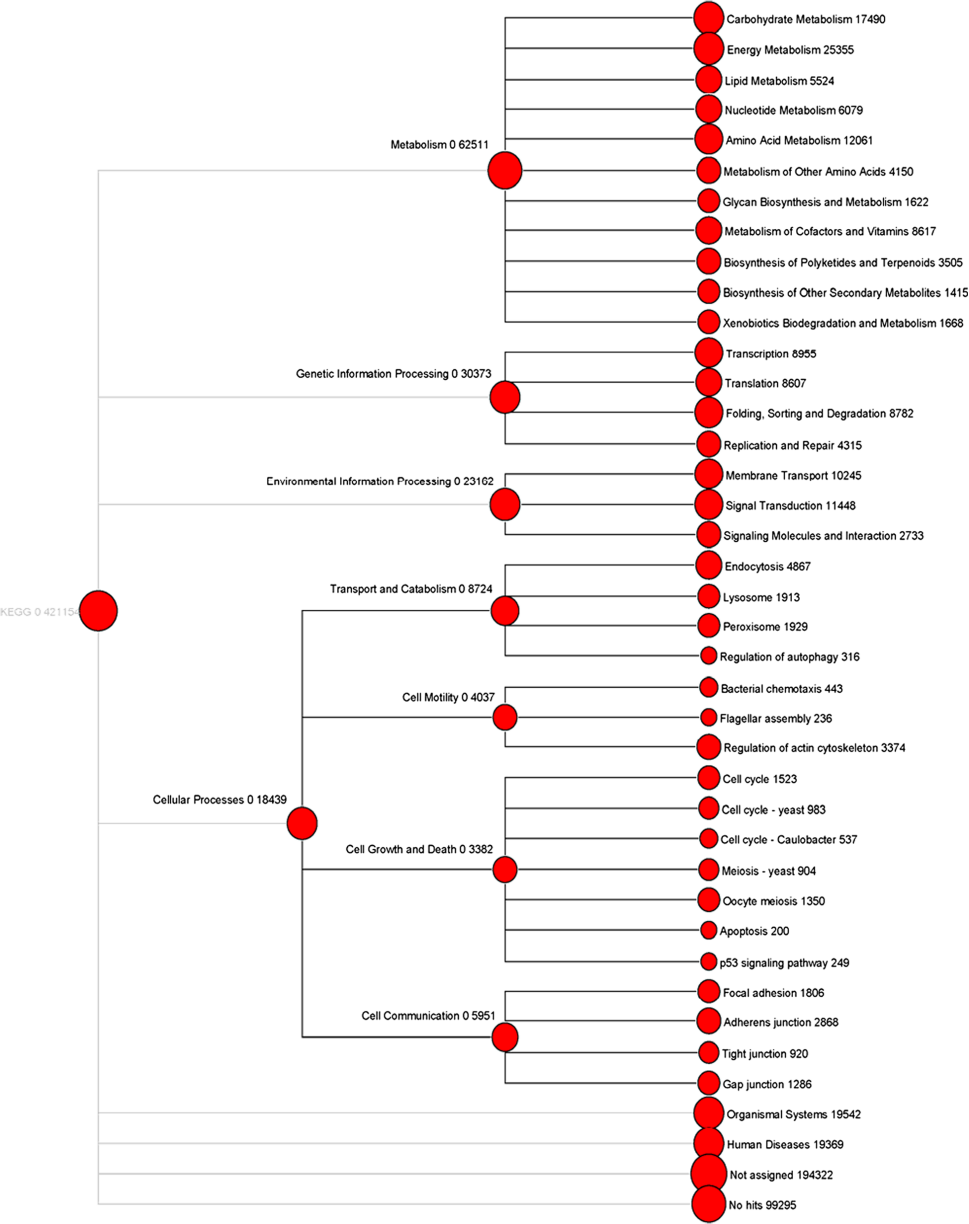
Metabolic pathway analysis using KEGG.

The Kyoto Encyclopedia of Genes and Genomes (KEGG) orthology (KO) is a classification system that provides an alternative functional annotation of genes based on their associated biological pathways (Figure 6). To reconstruct the metabolic pathways involved in *U. linza*, both reads and isotigs were assigned to the KEGG. In total 56,553 reads and 2,404 isotigs having enzyme commission (EC) numbers were assigned to 262 KEGG pathways. The highest numbers of reads were assigned to pathways on the second level of energy metabolism (25,355), carbohydrate metabolism (17,490), and immune systems (12,391) and on the third level of carbon fixation (9,356), ABC transporters (9,051) and photosynthesis (5,281). In contrast, the highest numbers of isotigs were assigned to pathways on the second level of carbohydrate metabolism (323), translation (311), and amino acid metabolism (254) and on the third level of ribosome (108), spliceosome (91), and purine metabolism (63) (Additional file 3: Table S3). The reason for the difference between reads and isotigs was that only a fraction of reads could be assembled into isotigs, and those unassembled reads may also have functional implications. Read-based KEGG annotation also can be used to estimate the expression abundance of certain KEGG functions. Pathways that possessed the highest number of isotigs were similar to those in *C. reinhardtii*, *V. carteri* and *P. patens*, but different from *Arabidopsis thaliana* on the third level.

### Photoprotection mechanisms in *U. linza*

In taxa of photosynthetic eukaryotes, the thylakoid membrane-integral light-harvesting complex (LHC) antenna systems, especially LHCII in Photosystem II, play important roles in regulating energy flow to reaction centers [45]. The LHC systems are generally used to harvest and transfer excitation energy into the reaction centers to drive photosynthesis. However, under excess light conditions, they undergo a conformational change and activate a quenching state for energy dissipation, termed non-photochemical quenching (NPQ) [46,47].

The light-harvesting antenna complex in PSII consists of two types of proteins: major trimer-forming LHCII antenna proteins and minor LHCII proteins. In land plants, major LHCII proteins are encoded by multiple Lhcb1, Lhcb2 and Lhcb3 genes [48], but in green algae, the major LHCII proteins encoded by Lhcbm genes showed great diversity [48-51]. Nineteen Lhcbm-encoding genes have been found in the transcripome of *U. linza*, and of these, the five genes that had full-length sequences were selected for phylogenetic tree construction. The phylogenetic tree shows that the major LHCII genes have undergone significant divergence in different organisms; this may be an adaptation mechanism to optimize light-harvesting capacity and acclimation to environmental conditions. Three minor PSII antenna have been found in land plants, namely CP26 (Lhcb5), CP29 (Lhcb4), and CP24 (Lhcb6); all of these are located at the interface between the major LHCII proteins and PSII [52,53]. The minor proteins CP26 and CP29 have been shown to play direct roles in NPQ in land plants [54]. The CP24 protein evolved differently and more recently in land plants as it is absent in all green algae [49,50]. In *U. linza*, only the CP26 and CP29 proteins were found as expected. From the phylogenetic tree, it can be seen that CP26 and CP29 are found in all green plant groups examined and evolved distinctly to the major LHCII proteins, which were present prior to diversification of the major lineages (Additional file 4: Figure S1). The conserved nature and distribution of the minor LHCII proteins suggests that they have a significant function.

**Figure 7:**
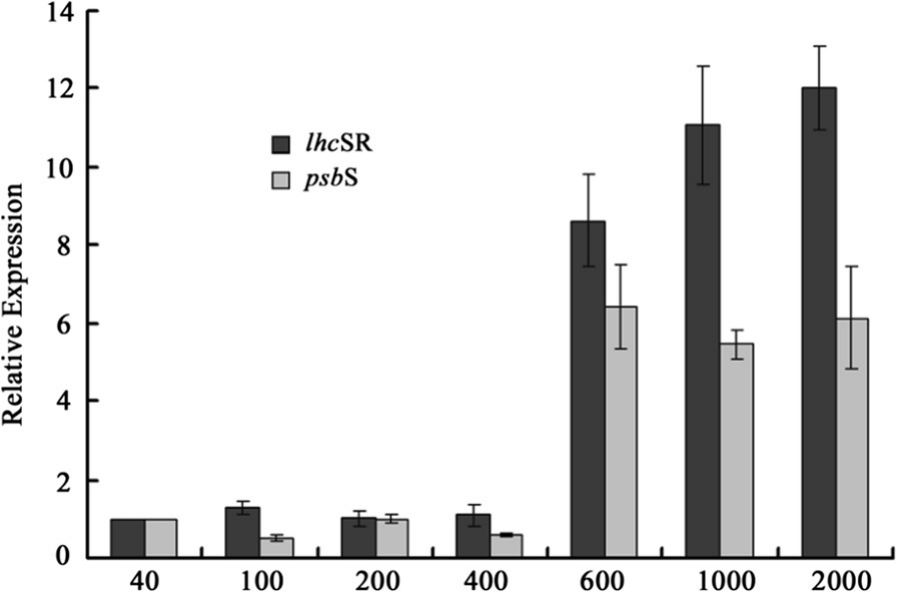
**Relative mRNA expression of *lhc*SR and *psb*S in *U. linza* exposed to 40-2000 μmol photons m**^**−2 **^**s**^**−1**^** for 1 h. Data were means ± SD (n=3).**

We also found the co-expressed *Lhc*SR and *Psb*S genes, which are photoprotection-related LHC genes. The PsbS protein was recently implicated in NPQ in land plants [55-57]. PsbS was not the site of quenching itself; instead it acts as a pH-dependent trigger to activate the quenching sites by regulating the organization of LHCII antenna system with the PSII core complex [58-61]. However, in green algae, such as *Chlamydomonas*, no accumulation of the PsbS protein could be detected, although the genes are structurally similar to PsbS [50,62]. The lack of the PsbS and CP24 proteins in green algae implied that the mechanistic basis of NPQ in these groups is likely different. In fact, the stress-related LHC member LhcSR proteins in green algae have been shown to participate in NPQ, likely indicating an early photoprotective mechanism [63]. Furthermore, the LhcSR3 protein in *Chlamydomonas* appears to merge both pH-sensing and energy-quenching functions in plants, which are accomplished by PsbS and monomeric Lhcb proteins, respectively [64]. Among the land plants, the mechanism of NPQ was unique in the moss, *P. patens*, where both the LhcSR and PsbS proteins are active [65]. The mechanism for NPQ was hypothesized to have evolved from being LhcSR-based to being PSBS-based during the transition to land colonization [63,65]. However, the transcription profiles of both *lhc*SR and *psb*S genes in *U. linza* indicate that the PsbS-based NPQ mechanism emerged before the land colonization. In addition, we found that both the *psb*S and *lhc*SR genes were induced by high light (>600 μmol photons m^−2^ s^−1^) and the expression patterns of these two genes were consistent (Figure 7), which indicates that both PsbS and LhcSR are active in photoprotection of *U. linza*. This result also suggests that the photoprotective mechanism in *Ulva* may differ from that of unicellular green algae. A potential explanation for this difference is that *Ulva* must cope with dramatic changes in the intensity and spectral quality of light in rocky coastal ecosystems. *U. linza* is a photophilous alga commonly distributed in shallow and horizontal habitats, like sun-exposed rocky shores. The light irradiance varies more dramatically for the algae than for land plants because of the morning and evening tides.

### Carbon fixation pathway

Besides photoprotection mechanisms, efficient carbon fixation is essential for photosynthesis, and as such likely to facilitate the colonization of coastal ecosystems and the accumulation of high *U. linza* biomass. Aquatic photosynthetic organisms are exposed to dramatic changes in the supply of dissolved inorganic carbon (Ci; CO_2_ and/or HCO^3-^). To acclimate to the Ci-limiting environmental conditions, they have developed a carbon concentrating mechanisms (CCM) to optimize the photosynthesis rate under the CO_2_-limiting condition [66,67]. At least one α-carbonic anhydrase (CA) with targeting signals for the chloroplast lumen was found in *U. linza*. Lumenal-targeted αCAs are well known from *C. reinhardtii* and higher plants and are essential for growth under ambient CO_2_ concentrations [68]. Thus, we predicted that the α-CA-based CCM type found in *U. linza* would show a strong similarity to the CCM in *C. reinhardtii*[69]. Few genes common to organisms that actively or passively enhance Ci influx in CCMs were found in *U. linza*. Only a putative soluble protein, LciB, was identified as a candidate for a chloroplast Ci transporter, and no Ci membrane transporter candidate was found. Five low/limited CO_2_-induced proteins were identified, including two regulatory factors (CCM1 and LCR1) that control the expression of the CO_2_-related genes.

C4-photosynthesis is an important feature in some higher plants in tropical environments, as it helps them cope well with high light intensities, high temperatures, and dryness [70,71]. Some intermediate products of the C4 carbon-fixation pathway have been detected in diatoms, brown algae, green algae and red algae [72-77]. The C4-like CCM mechanism in green algae of *O. tauri*[38] and *Micromonas*[39] may provide them an ecological advantage in the CO_2_-limiting conditions of phytoplankton blooms. In *Ulva prolifera*, coexistence of C3 and C4-like pathway may contribute to its wide distribution and forming blooms [78]. In *U. linza*, genes encoding all of the enzymes required for C4 photosynthesis were identified implying a putative C4-like CCM mechanism (Additional file 5: Table S4).

### Transporters

Transporters are mainly responsible for the acquisition, redistribution and compartmentalization of organic nutrients and inorganic ions, as well as for the efflux of toxic compounds and metabolic end products. A total of 114 transport members were found in the *U. linza* transcriptome, and the complement resembles that of both green algae and land plants. The ATP-binding cassette (ABC) family (36 members) and the P-type adenosine triphosphatase (ATPase) family (12 members) are large compared to other transporters. One homologue of a plant-specific peroxisomal ABC transporter was found in *Ulva*, but is absent in the genomes of other green algae. *Ulva* is known for its ability to take up inorganic nutrients and use them to grow rapidly, which allows it to be successful in nitrogen-rich areas and form the green tides [8,11]. The transcriptome encodes at least 19 transporters for essential inorganic nutrients (i.e., nitrogen, phosphorus, and sulphur). *U. linza* obtains nitrogen in at least three different ways, and ammonium is the best nitrogen source for *Ulva* growth, followed by urea and nitrate [79]. The complete set of genes allowing transport and assimilation of these substrates include six ammonium transporters, three high-affinity nitrate transporters, and two urea transporters. The high number of ammonium transporters indicates that *Ulva* is a strong competitor for this resource, which is very efficiently pumped from surrounding seawater and is probably the privileged nitrogen source for *Ulva*. The inorganic nutrient transporters found in *Ulva* showed multiple origins. The unicellular green algae *O. tauri* has two prokaryote-like ammonium transporters, and one was found in *U. linza* (Additional file 6: Figure S2). All of the three phosphate transporters found in *U. linza* are absent in higher land plants, two occur only in green algae, and the third one is present in moss (Additional file 7: Figure S3). These results suggest that the phosphate transporting mechanism in *Ulva* may be old and associated with aquatic environments and that it was lost during adaptation to land. Of the three sulfate transporters, two are in the Na^+^/SO_4_^2-^-family; neither of these is present in higher land plants but one is present in moss. The third one is conserved in both green algae and high plants (Additional file 8: Figure S4).

### Cytochrome P450 oxidoreductases

Eukaryotic cytochrome P450s are oxidoreductases, most of which catalyze NADPH- and O_2_-dependent hydroxylation reactions [80]. Plant P450s participate in a myriad of biochemical pathways, including those devoted to the synthesis of plant products and plant growth regulators [81]. The complexity of the P450 family is reflected in the evolution of the metabolic complexity of organisms. *U. linza* has at least 25 cytochrome P450s, which is a higher number than that found in other algae (eg. the unicellular green alga *Chlamydomonas* (13) and brown alga *Ectocarpus* (12)), but a lower number than that found in land plants (e.g., *Physcomitrella* (71) and *Arabidopsis* (286)). Nearly half of the P450s in *U. linza* are conserved in algae (even cyanobacteria) (Additional file 9: Figure S5). These P450s likely originated in algae and may be related to acclimatization to the aquatic environment. The role of these conserved P450s is poorly understood. A few of members of the CYP97 family are known to be involved in accumulating secondary ketocarotenoids in response to stress in terrestrial plants [82,83]. Some P450s genes in *U. linza* are homologous to those in fungus, land plants, and even animals but are absent in unicellular green algae. We predicted that these genes may function in allelopathy in *U. linza*, thus allowing it to produce toxic substances for defense against herbivory or other phototrophic organisms that compete for light and nutrients [84-86]. *U. linza* is known to exhibit strong allelopathy: as one of the most common green tide macroalgal species, it has to outcompete other coastal primary producers and establish dominant populations in a short time [87].

### Stress-related proteins

Glutamate dehydrogenase (GDH) catalyzes the reversible oxidative deamination of glutamate to α-ketoglutarate and ammonia. In plants, the enzyme can work in either direction depending on environment and stress [88,89]. It links the two fundamental metabolic processes involving carbon and nitrogen within a cell, and transgenic plants expressing microbial GDHs exhibit improved tolerance to herbicides, water deficits, and pathogen infections [89,90]. Two GDH genes were found in *Arabidopsis*, and only one was found in *Chlamydomonas*. In contrast, eight putative GDH genes were found in *U. linza*. Seven of these GDH genes have similarities to those found in bacteria, fungus, and even animals (Additional file 10: Figure S6). Lateral gene transfer has been shown to play a significant role in the evolution of the GDH genes [91]. Thus, we predicted that the diverse GDH genes in *Ulva* may have been obtained recently by gene transfer and function in maintaining a balance between carbon and nitrogen metabolites and enhancing the stress tolerance.

Reactive oxygen species generated during photosynthesis are scavenged by superoxide dismutases (SODs), ascorbate peroxidase (APX), and catalase (CAT) in plants [92-94]. Four putative APX genes were found in *U. linza*, which is more than has been found in *C. reinhardtii* (2), *V. carteri* (3), and *P. patens* (3) but less than in *A. thaliana* (6). All four APX genes are conserved in green algae and land plants (Additional file 11: Figure S7). In contrast, the two CAT genes in *U. linza* show higher similarities to those in fungi and bacteria than to those in green algae, and they have no homologous genes in land plants (Additional file 12: Figure S8). The APX and CAT genes, which are involved in scavenging reactive oxygen, may have evolved in different ways. Two Fe-type and one Mn-type SODs were found in *U. linza*, but no Cu/Zn-type or Ni-containing SOD was found. However, a Cu/Zn-type SOD was found to be expressed in *Ulva fasciata* in response to copper stress [95]. We predicted that Fe-type and Mn-type SODs may play dominant roles in response to broad stress conditions, such as high salt, high light, and high temperature conditions. In contrast, the Cu/Zn-type SOD may be related only to heavy metal stresses.

Heat-shock proteins (Hsps) constitute a major class of stress-response proteins that mainly assist in protein refolding under stress conditions. Thirty-two Hsp genes were found in *U. linza* from four conserved families: Hsp70, Hsp90, Hsp100, and low-molecular-mass Hsps (sHsps). In *U. linza*, 8 sHsps belong to the Hsp20 family, and these Hsp20 proteins show high similarities to prokaryotes as to green algae and plants (Additional file 13: Table S5; Additional file 14: Figure S9). Similar observations were also made in terrestrial plants, where the sHsps are highly diverse, such as in *Arabidopsis*, 13 different sHsps are grouped into six classes [96]. In contrast to Hsp20 proteins, most members of the Hsp70, Hsp90 and Hsp100 families found in *U. linza* were conserved well in eukaryotic algae and plants, with the exception of only three (Additional file 13: Table S5). Compared to other unicellular green algae, number of Hsp90 and Hsp100 proteins was increased in *U. linza*, especially members of the Hsp90 family (Additional file 15: Table S6). The Hsp90 family is distinct in that most of its known substrates are signal transduction proteins, such as steroid hormone receptors and signaling kinases [97], which may play a key role in signal transduction networks, protein degradation and protein trafficking. Thus, the increasing number of Hsp90 members indicates that *U. linza* may possess a Hsp/chaperone network that is more complicated than that found in unicellular algae, and it may be related to the acclimation of *U. linza* to the intricate environment of the intertidal zone.

## Conclusions

The green algal genus Ulva Linnaeus (Ulvaceae, Ulvales, Chlorophyta) is well known for its wide distribution in marine, freshwater, and brackish environments throughout the world. The *Ulva* species are also highly tolerant of variations in salinity, temperature, and irradiance and are the main cause of green tides, which can have deleterious ecological effects. However, only limited genomic information is currently available in this non-model and ecologically important species. The transcriptome of *U. linza* uncovers some of potential genomic mechanisms that might explain its ability to rapidly and successfully colonize the coastal ecosystems, including the specific photoprotective mechanism based on both *Lhc*SR and *Psb*S; development of C4-like carbon-concentrating mechanisms; muti-origin transporters for essential inorganic nutrients; multiple and complex P450s, glutamate dehydrogenase, superoxide dismutases, ascorbate peroxidase, catalase and heat-shock proteins that are related to stress resistance.

## Methods

### Sampling and culture conditions

The *Ulva linza* samples were collected in May of 2010 from intertidal zone 20 (35°35´N, 119°30´E) of Zhanqiao Wharf, Qingdao, China. In the laboratory, the intact 21 samples were washed several times with sterile seawater, sterilized with 1% sodium hypochlorite for 2 min, and then rinsed with autoclaved seawater. The sterilized material was then placed into an aquarium containing enriched seawater and which was aerated and maintained at 15°C under a 12:12 h LD photoperiod with 120 μmol photons m^−2^ s^−1^ provided by cool white fluorescent tubes.

### Library preparation and 454 sequencing

The samples of *Ulva linza* were subjected to different stress conditions: low temperature (6°C, 2 h), high temperature (42°C, 1 h), high light (400 μmol photons m^−2^ s^−1^, 1 h), high salt (93‰, 3 h) and UV-B stress (60 μw cm^-2^, 3 h). All the treated samples were frozen in liquid nitrogen, and total RNA was extracted using the TRIzol reagent (Invitrogen) according to the manufacturer’s recommendations. The mRNA was purified from the total RNA using the Oligotex mRNA Midi Kit (QIAGEN, Germany). Double-stranded cDNA was then synthesized using the SMART cDNA Library Construction kit (Clontech, USA) by following the manufacturer’s protocol. The cDNA was purified using Qiagen QIAquick PCR purification spin columns. Normalization was performed using the TRIMMER cDNA normalization kit (Evrogen) to decrease the prevalence of abundant transcripts before sequencing, and the quality of cDNA was checked using the Agilent 2100 Bioanalyzer. A half-plate sequencing run was performed at the Tianjin Biochip Corporation following the manufacturer’s protocols. Raw data has been submitted to the Status of the NCBI Sequence Read Archive (http://www.ncbi.nlm.nih.gov/Traces/sra_sub/sub.cgi?).

### *De novo* assembly

The “-cdna” option of the latest release of Roche 454’s Newbler (version 2.5p1) was used to assemble transcriptome. Newbler creates isotigs out of contigs that are consistently connected by a subset of reads. Each isotig corresponds to an alternative transcript, and any contigs or isotigs that share any read overlaps are put into the same isogroup.

### Functional annotation

We used local BLASTX to align 454 reads to the GenBank nonredundant protein (nr) database using an E value threshold of 10^-2^. The unigene sequences were also translated into proteins using ESTScan and the translated protein sequences were then compared to InterPro and pfam domain databases. The gene ontology (GO) terms were assigned to each unigene based on the GO terms annotated to its corresponding homologues in the UniProt database, and based on the InterPro and Pfam domains using interpro2go and pfam2go mapping files provided by the GO website, respectively [98]. Then, the MEGAN v4.0 software [99] was used to assign the annotated reads to KEGG pathways [100]. The number of reads assigned to each KEGG functional category can be used to evaluate gene quantities and expression levels.

### Real-time RT-PCR analysis

Real-time RT-PCR approach was employed to analyze the expression of *lhc*SR and *psb*S genes under different stress conditions. The primers for real-time RT-PCR analysis in this experiment are shown in Additional file 16: Table S7. The different light intensity treatments were provided at 120, 240, 400, 500, and 600 μmol photons m^−2^ s^−1^ and lasted 1 h at 15°C. For each condition, three biological replicates were conducted. The expression of the genes under different conditions was measured by real-time PCR. The qPCR was performed with the ABI StepOne plus Real-Time PCR System (Applied Biosystems, USA) and performed using SYBR Green fluorescence (Takara) according to the manufacturer’s instructions. The RNA levels were expressed relative to those of the 18S rDNA sequence. The thermal profile for real-time PCR was 30 s followed by 40 cycles at 95°C for 5 s, 55°C for 10 s and 72°C for 30 s. Dissociation curve analysis of the amplification products was performed at the end of each PCR reaction to confirm that only one specific PCR product was amplified and detected. The qPCRs were performed in triplicate for each sample. The 2^-ΔΔCT^ method [101] was used to analyze the quantitative real-time PCR data.

## Competing interests

The authors declare that they have no competing interests.

## Authors’ contributions

XZ, NY, and ZZ conceived and designed the study; SM, XF and JX conducted culturing and molecular biology laboratory work; XZ, CL and DX undertook data analyses; all authors contributed to the manuscript. All authors read and approved the final manuscript.

## Supplementary Material

Additional file 1 **Table S1.** Annotation of *Ulva* genes occurring in *Chlamydomonas reinhardtii*, but not in *Ostreococcus tauri*. Click here for file

Additional file 2 **Table S2.** Eighteen *Ulva* special isotigs conserved in land plants. Click here for file

Additional file 3 **Table S3.** a. Comparison of *Ulva* KEGG annotation analyzed by Reads and Isotigs respectively. b. Comparison of KEGG annotation among *Ulva linza* (isotigs), *Chlamydomonas reinhardtii, Volvox carteri, Physcomitrella patens and Arabidopsis thaliana* on three different levels. Click here for file

Additional file 4 **Figure S1.** Phylogenetic analysis of Lhcb proteins in six organisms. The phylogenetic tree was constructed by the neighbor-joining algorithm of the MEGA 4.0 program. A total of 1,000 bootstrap replicates were performed. Bootstrap values (1000 replicates) >50% are indicated on the branch. Click here for file

Additional file 5 **Table S4.** Putative genes encoding the enzymes required for a C4-like CCM. Click here for file

Additional file 6 **Figure S2.** Phylogenetic analysis of putative ammonium transporters in *U. linza*. The phylogenetic tree was constructed by the neighbor-joining algorithm of the MEGA 4.0 program. A total of 1,000 bootstrap replicates were performed. The prokaryote-like ammonium transporters found in *U. linza* was showed in red color. Click here for file

Additional file 7 **Figure S3.** Phylogenetic analysis of putative phosphate transporters in *U. linza*. The phylogenetic tree was constructed by the neighbor-joining algorithm of the MEGA 4.0 program. A total of 1,000 bootstrap replicates were performed. Click here for file

Additional file 8 **Figure S4.** Phylogenetic analysis of putative sulfate transporters in *U. linza*. The phylogenetic tree was constructed by the neighbor-joining algorithm of the MEGA 4.0 program. A total of 1,000 bootstrap replicates were performed. Click here for file

Additional file 9 **Figure S5.** Phylogenetic analysis of putative P450s in *U. linza*. The phylogenetic tree was constructed by the neighbor-joining algorithm of the MEGA 4.0 program. A total of 1,000 bootstrap replicates were performed. Click here for file

Additional file 10 **Figure S6.** Phylogenetic analysis of putative Glutamate dehydrogenases (GDH) in *U. linza*. The phylogenetic tree was constructed by the neighbor-joining algorithm of the MEGA 4.0 program. A total of 1,000 bootstrap replicates were performed. Click here for file

Additional file 11 **Figure S7.** Phylogenetic analysis of putative ascorbate peroxidase (APX) genes in *U. linza*. The phylogenetic tree was constructed by the neighbor-joining algorithm of the MEGA 4.0 program. A total of 1,000 bootstrap replicates were performed. Click here for file

Additional file 12 **Figure S8.** Phylogenetic analysis of putative catalase (CAT) genes in *U. linza*. The phylogenetic tree was constructed by the neighbor-joining algorithm of the MEGA 4.0 program. A total of 1,000 bootstrap replicates were performed. Click here for file

Additional file 13 **Table S5.** Putative Heat-shock proteins found in U. linza. Three genes encoding Hsp70, Hsp90 and Hsp100 that not conserved in green algae and plants were shown in yellow color. Click here for file

Additional file 14 **Figure S9.** Phylogenetic analysis of putative Hsp20 proteins in *U. linza*. The phylogenetic tree was constructed by the neighbor-joining algorithm of the MEGA 4.0 program. A total of 1,000 bootstrap replicates were performed. Click here for file

Additional file 15 **Table S6.** Comparison of Heat-shock proteins among *Ulva linza*, *Micromonas* sp. RCC299, *Ostreococcus tauri, Chlorella variabilis* NC64A*, Chlamydomonas reinhardtii, Volvox carteri* and *Arabidopsis thaliana*. Click here for file

Additional file 16 **Table S7.** Primers used in the real-time RT-PCR analysis. Click here for file
