# Supplementary material for: De novo sequencing and analysis of the Ulva linza transcriptome to discover putative mechanisms associated with its successful colonization of coastal ecosystems
Source: BMC Genomics. 2012 Oct 25;13:565. doi: 10.1186/1471-2164-13-565 (PMC3532339; doi:10.1186/1471-2164-13-565)
Supplement: Additional file 2 — Table S2. Eighteen Ulva special isotigs conserved in land plants. [file 1471-2164-13-565-S2.doc]

**Additional file 2 Table S2** Eighteen *Ulva* special isotigs conserved in land plants

|  | Name | Putative function | E value | Best blast |
| --- | --- | --- | --- | --- |
| 1 | contig00217 | NBS-LRR disease resistance-like protein | 5e-05 | *Populus trichocarpa*  EEF09283.1 |
| 2 | contig00259 | cc-nbs-lrr resistance protein | 1e-16 | *Populus trichocarpa*  EEE75263.1 |
| 3 | isotig00489 | NBS-LRR resistance protein | 2e-04 | *Rosa hybrid cultivar*  ACO24729.1 |
| 4 | isotig03038 | glycine-rich protein | 5e-09 | *Arabidopsis thaliana*  [AAL62398.1](http://www.ncbi.nlm.nih.gov/protein/18252943?report=genbank&log$=protalign&blast_rank=1&RID=0WH59TKG01N) |
| 5 | contig03228 | ABC transporter G family member | 3e-04 | *Medicago truncatula*  AES73687.1 |
| 6 | isotig03300 | N-acetylglucosaminyl transferase | 5e-06 | *Ricinus communis*  EEF37506.1 |
| 7 | isotig03791 | disease resistance protein (TIR-NBS-LRR class) | 3e-25 | *Arabidopsis thaliana*  [AAD25848.3](http://www.ncbi.nlm.nih.gov/protein/20198191?report=genbank&log$=protalign&blast_rank=1&RID=0WJF9YVF01S) |
| 8 | isotig04241 | disease resistance protein, putative | 6e-37 | *Arabidopsis thaliana* BAE98852.1 |
| 9 | isotig04741 | AtRLP4 (Receptor Like Protein 4) | 2e-08 | *Arabidopsis thaliana*  AEE30960.1 |
| 10 | isotig04834 | isoaspartyl peptidase/L-asparaginase 3 | 7e-84 | *Glycine max*  XP_003527550.1 |
| 11 | isotig07516 | phospholipase D alpha | 1e-79 | *Citrus sinensis*  ACA49723.1 |
| 12 | isotig10008 | zinc finger (C2H2 type) family protein | 1e-05 | *Arabidopsis thaliana*  AED91992.1 |
| 13 | contig06251 | Transcription elongation regulator | 2e-06 | *Medicago truncatula*  AES89399.1 |
| 14 | contig00217 | cc-nbs-lrr resistance protein | 2e-06 | Populus trichocarpa  [EEF09283.1](http://www.ncbi.nlm.nih.gov/protein/222872152?report=genbank&log$=protalign&blast_rank=1&RID=0WTSJXSV012) |
| 15 | contig00257 | hypothetical protein | 1e-10 | Sorghum bicolor  EER93136.1 |
| 16 | isotig06105 | hypothetical protein | 8e-14 | Selaginella moellendorffii  EFJ31667.1 |
| 17 | isotig09080 | hypothetical protein | 2e-59 | Glycine max  XP_003556363.1 |
| 18 | isotig09801 | unknown protein [Arabidopsis thaliana] | 3e-11 | Glycine max  [XP_003542739.1](http://www.ncbi.nlm.nih.gov/protein/356548702?report=genbank&log$=protalign&blast_rank=1&RID=0WV7BKAG016) |
