# Supplementary material for: De novo sequencing and analysis of the Ulva linza transcriptome to discover putative mechanisms associated with its successful colonization of coastal ecosystems
Source: BMC Genomics. 2012 Oct 25;13:565. doi: 10.1186/1471-2164-13-565 (PMC3532339; doi:10.1186/1471-2164-13-565)
Supplement: Additional file 3 — Table S3. a. Comparison of Ulva KEGG annotation analyzed by Reads and Isotigs respectively. b. Comparison of KEGG annotation among Ulva linza (isotigs), Chlamydomonas reinhardtii, Volvox carteri, Physcomitrella patens and Arabidopsis thaliana on three different levels. [file 1471-2164-13-565-S3.doc]

**Additional file3 Table S3a** Comparison of *Ulva* KEGG annotation analyzed by Reads and Isotigs respectively.

|  | First level | | | Second level | | | Third level | | |
| --- | --- | --- | --- | --- | --- | --- | --- | --- | --- |
| Reads | Metabolism | Genetic information processing | Environmental information processing | energy metabolism | carbohydrate metabolism | Immune systems | Carbon fixation | ABC transporters | Photosynthesis |
|  | 62,511 | 30,373 | 23,162 | 25,355 | 17,490 | 12,391 | 9,356 | 9051 | 5,281 |
| Isotigs | Metabolism | Genetic information processing | Cellular Processes | carbohydrate metabolism | Translation | Amino Acid Metabolism | Ribosome | Spliceosome | Purine metabolism |
|  | 1476 | 765 | 271 | 323 | 311 | 254 | 108 | 91 | 63 |

**Additional file3 Table S3b** Comparison of KEGG annotation among *Ulva linza* (isotigs), *Chlamydomonas reinhardtii, Volvox carteri, Physcomitrella patens and Arabidopsis thaliana* on three different levels.

|  | First level | | | Second level | | | Third level | | | |
| --- | --- | --- | --- | --- | --- | --- | --- | --- | --- | --- |
| *Ulva linza* | Metabolism  1476 | Genetic information processing  765 | Organismal Systems  330 | carbohydrate metabolism  323 | Translation  311 | Amino Acid Metabolism  254 | Ribosome  108 | Spliceosome  91 | RNA transport  65 | Purine metabolism  63 |
| *Chlamydomonas reinhardtii* | -  1865 | -  1081 | -  468 | -  391 | -  335 | -  292 | -  118 | Purine metabolism  123 | Spliceosome  94 | RNA transport  76 |
| *Volvox carteri* | -  1818 | -  927 | -  379 | -  395 | -  361 | -  308 | -  142 | Purine metabolism  112 | Spliceosome  101 | RNA transport  79 |
| *Physcomitrella patens* | -  3799 | -  1855 | -  1096 | -  1016 | -  862 | -  583 | -  460 | Purine metabolism  167 | RNA transport  161 | Spliceosome  156 |
| *Arabidopsis thaliana* | -  3946 | -  1605 | -  1123 | -  909 | -  648 | -  585 | -  298 | Plant hormone signal transduction  230 | Oxidative phosphorylation  162 | Protein export  143 |
