# Supplementary figures and images for: De novo sequencing and analysis of the Ulva linza transcriptome to discover putative mechanisms associated with its successful colonization of coastal ecosystems
Source: BMC Genomics. 2012 Oct 25;13:565. doi: 10.1186/1471-2164-13-565 (PMC3532339; doi:10.1186/1471-2164-13-565)

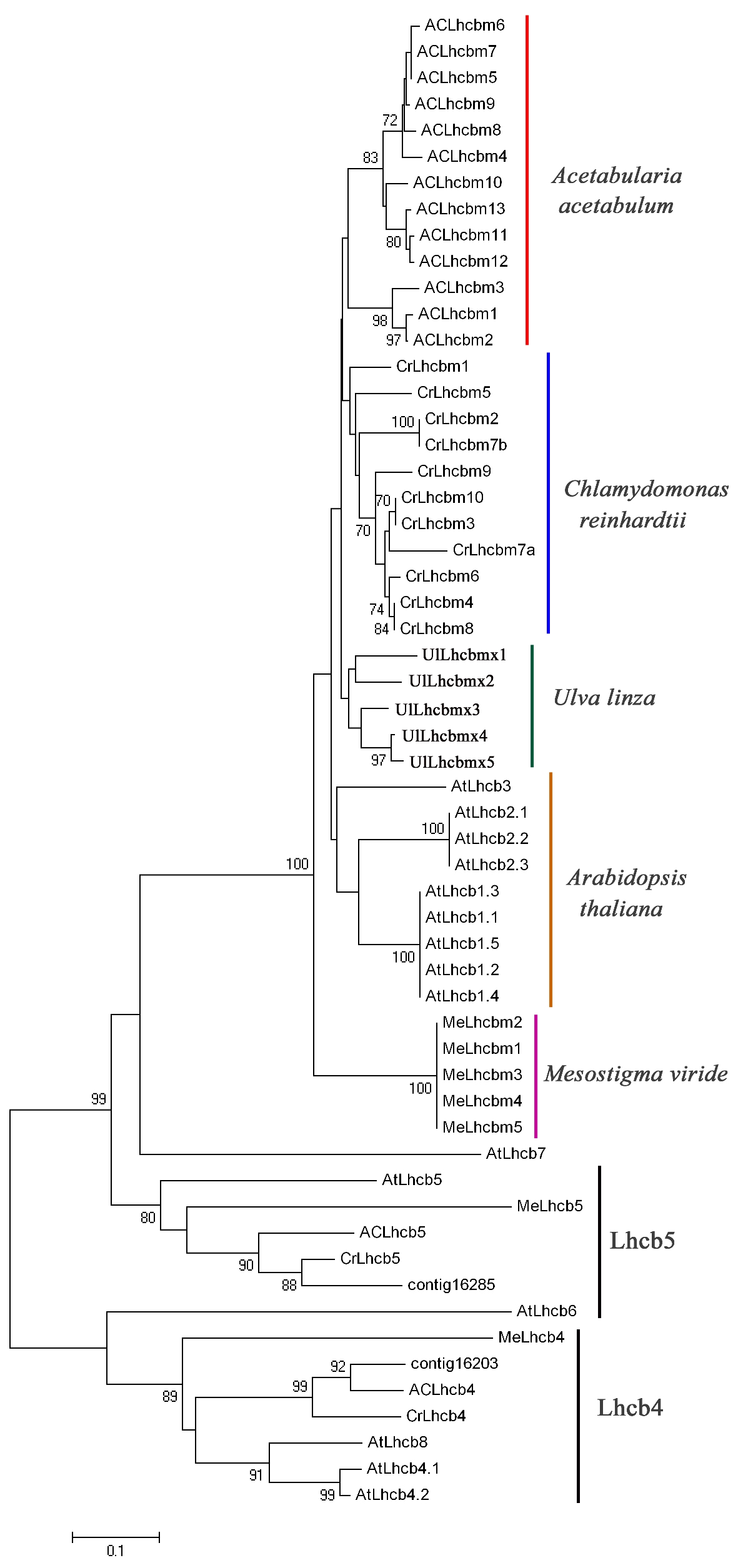

Supplement: Additional file 4 — Figure S1. Phylogenetic analysis of Lhcb proteins in six organisms. The phylogenetic tree was constructed by the neighbor-joining algorithm of the MEGA 4.0 program. A total of 1,000 bootstrap replicates were performed. Bootstrap values (1000 replicates) >50% are indicated on the branch. [file 1471-2164-13-565-S4.jpeg]

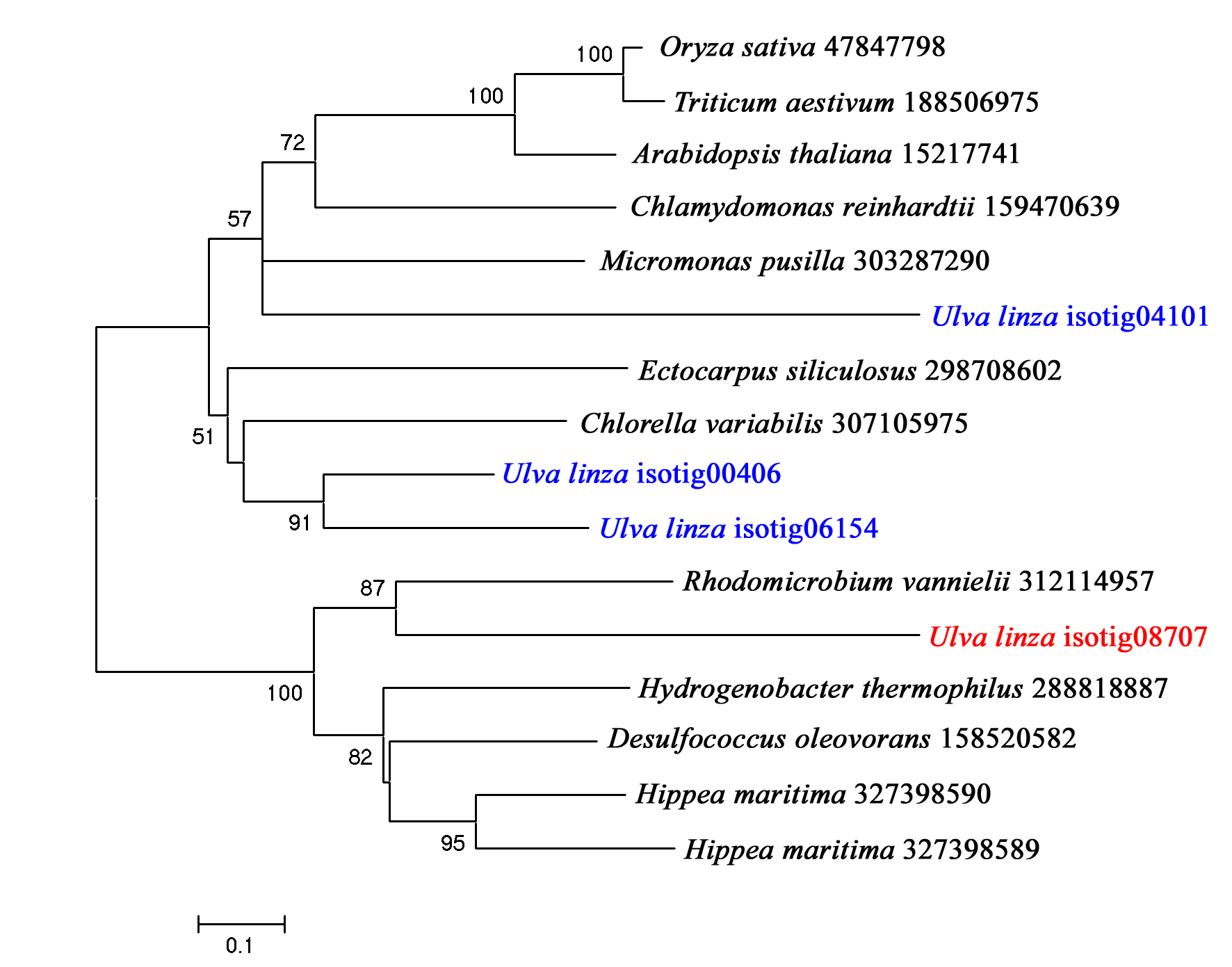

Supplement: Additional file 6 — Figure S2. Phylogenetic analysis of putative ammonium transporters in U. linza. The phylogenetic tree was constructed by the neighbor-joining algorithm of the MEGA 4.0 program. A total of 1,000 bootstrap replicates were performed. The prokaryote-like ammonium transporters found in U. linza was showed in red color. [file 1471-2164-13-565-S6.jpeg]

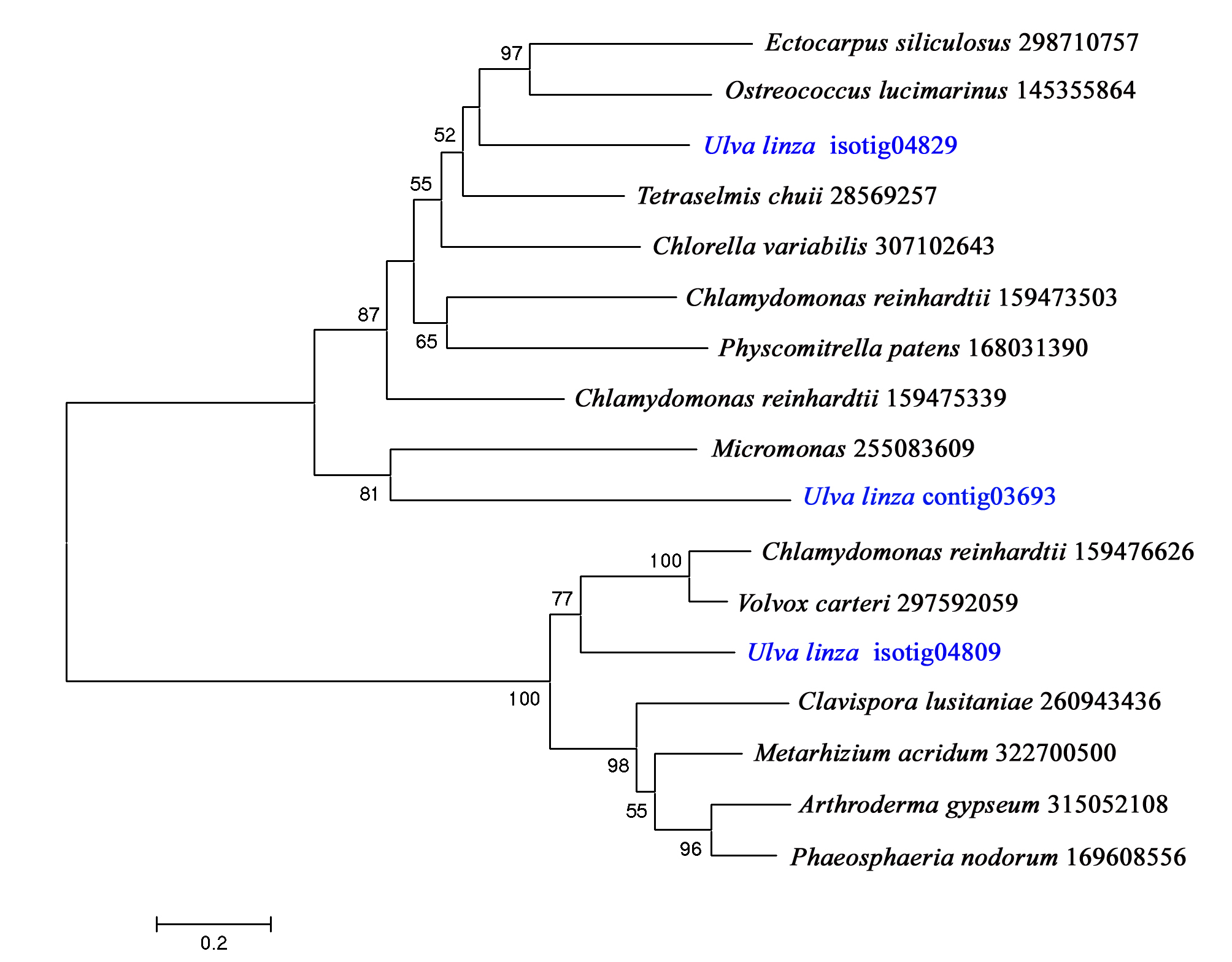

Supplement: Additional file 7 — Figure S3. Phylogenetic analysis of putative phosphate transporters in U. linza. The phylogenetic tree was constructed by the neighbor-joining algorithm of the MEGA 4.0 program. A total of 1,000 bootstrap replicates were performed. [file 1471-2164-13-565-S7.jpeg]

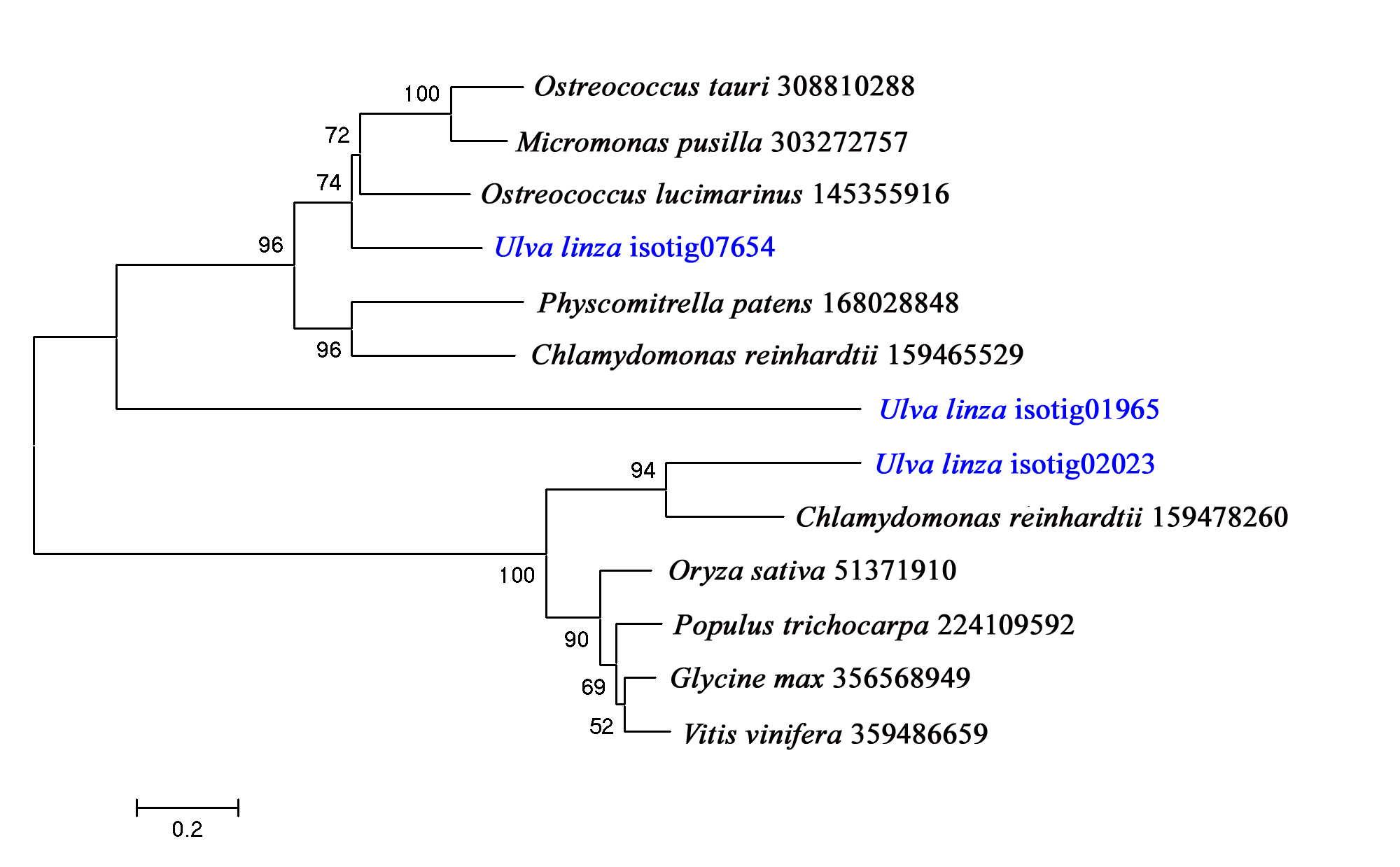

Supplement: Additional file 8 — Figure S4. Phylogenetic analysis of putative sulfate transporters in U. linza. The phylogenetic tree was constructed by the neighbor-joining algorithm of the MEGA 4.0 program. A total of 1,000 bootstrap replicates were performed. [file 1471-2164-13-565-S8.jpeg]

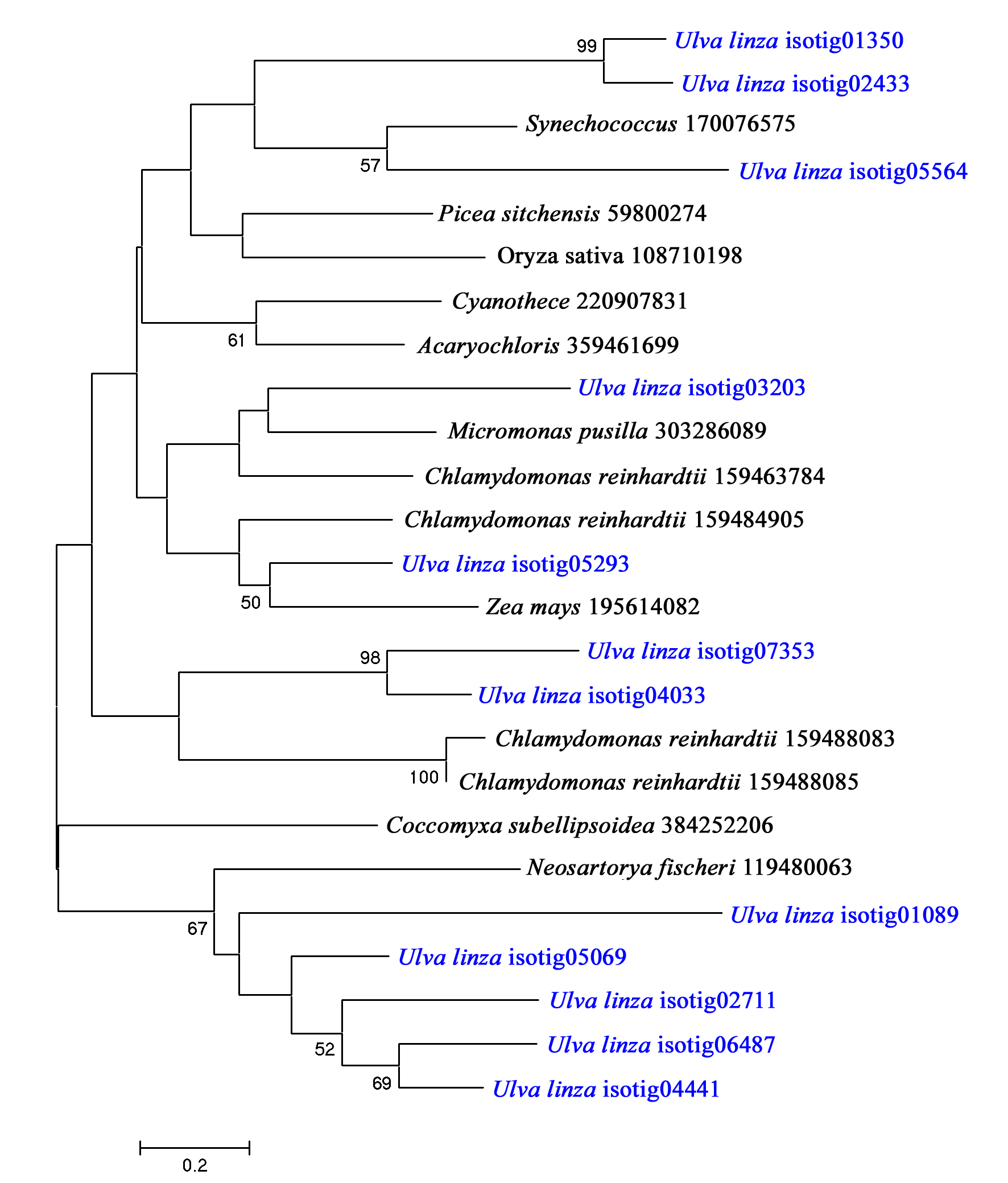

Supplement: Additional file 9 — Figure S5. Phylogenetic analysis of putative P450s in U. linza. The phylogenetic tree was constructed by the neighbor-joining algorithm of the MEGA 4.0 program. A total of 1,000 bootstrap replicates were performed. [file 1471-2164-13-565-S9.jpeg]

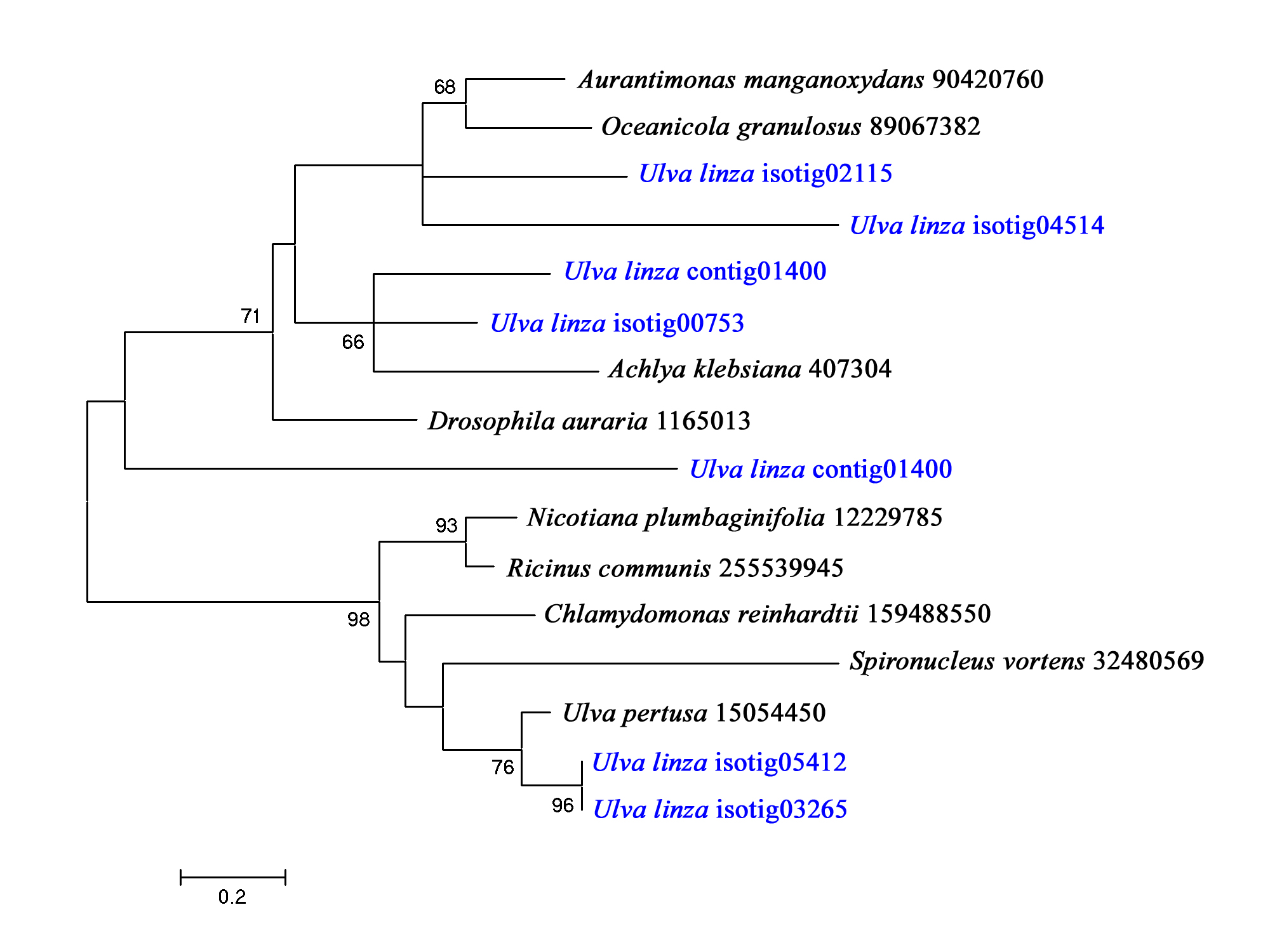

Supplement: Additional file 10 — Figure S6. Phylogenetic analysis of putative Glutamate dehydrogenases (GDH) in U. linza. The phylogenetic tree was constructed by the neighbor-joining algorithm of the MEGA 4.0 program. A total of 1,000 bootstrap replicates were performed. [file 1471-2164-13-565-S10.jpeg]

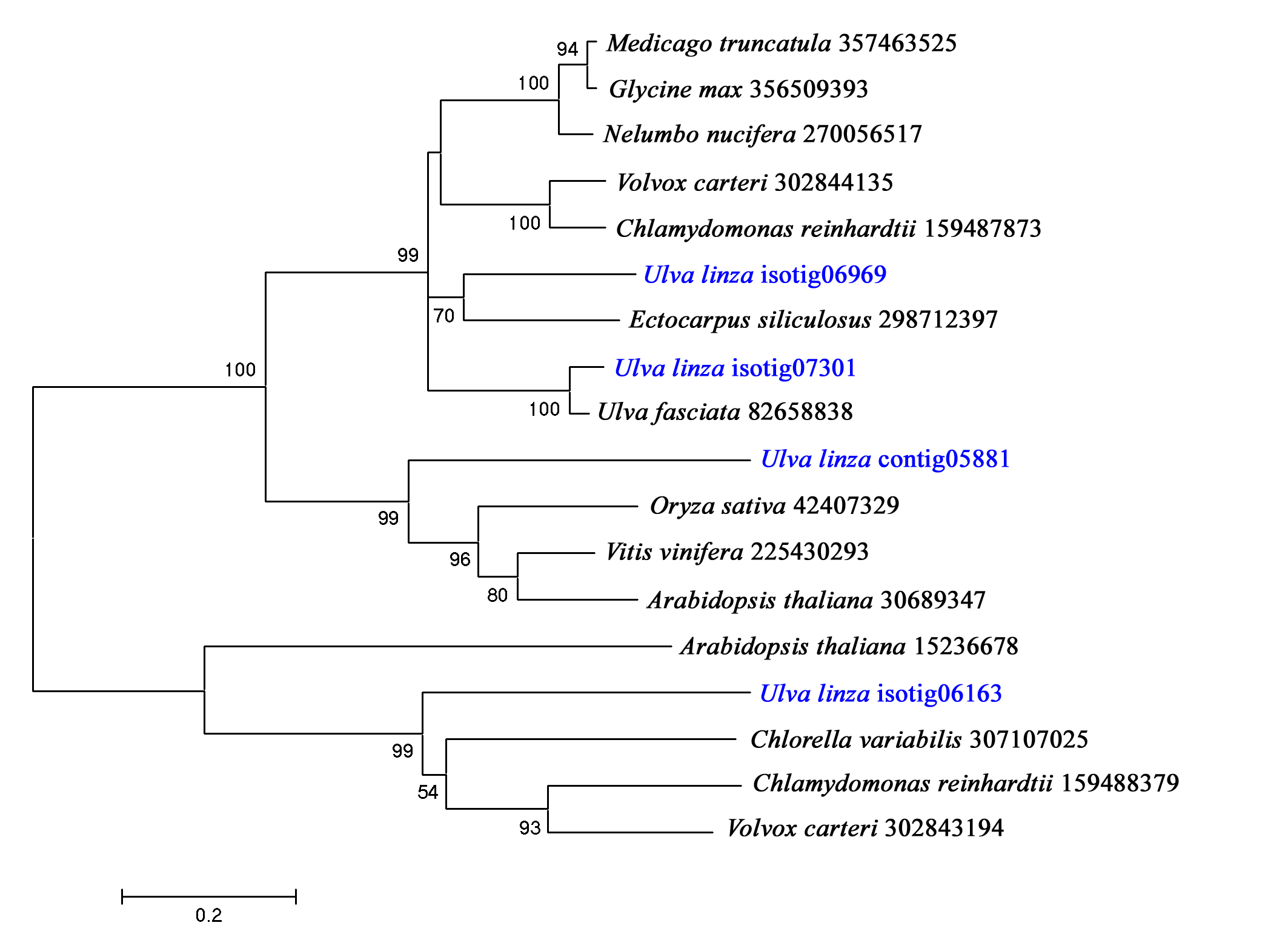

Supplement: Additional file 11 — Figure S7. Phylogenetic analysis of putative ascorbate peroxidase (APX) genes in U. linza. The phylogenetic tree was constructed by the neighbor-joining algorithm of the MEGA 4.0 program. A total of 1,000 bootstrap replicates were performed. [file 1471-2164-13-565-S11.jpeg]

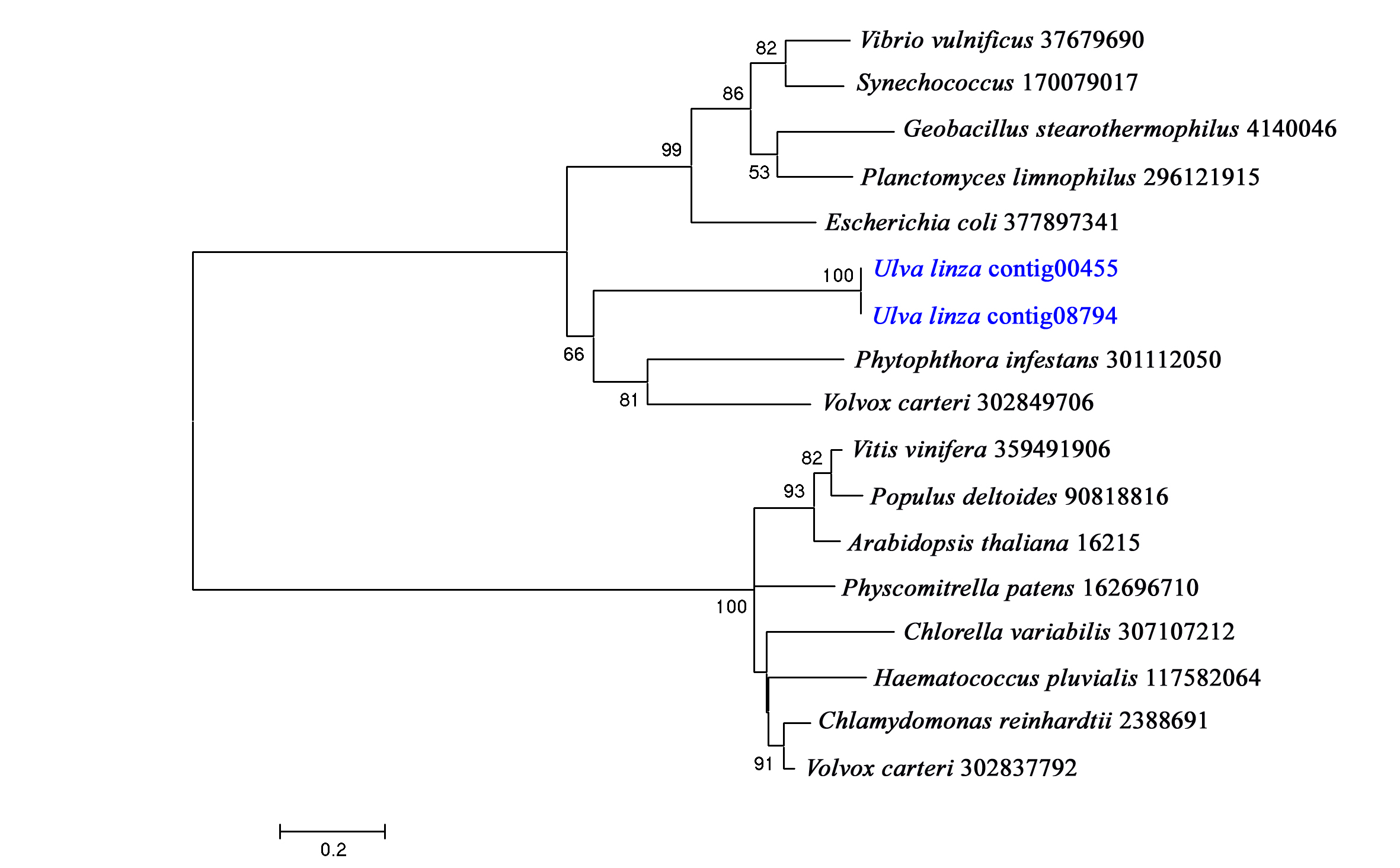

Supplement: Additional file 12 — Figure S8. Phylogenetic analysis of putative catalase (CAT) genes in U. linza. The phylogenetic tree was constructed by the neighbor-joining algorithm of the MEGA 4.0 program. A total of 1,000 bootstrap replicates were performed. [file 1471-2164-13-565-S12.jpeg]

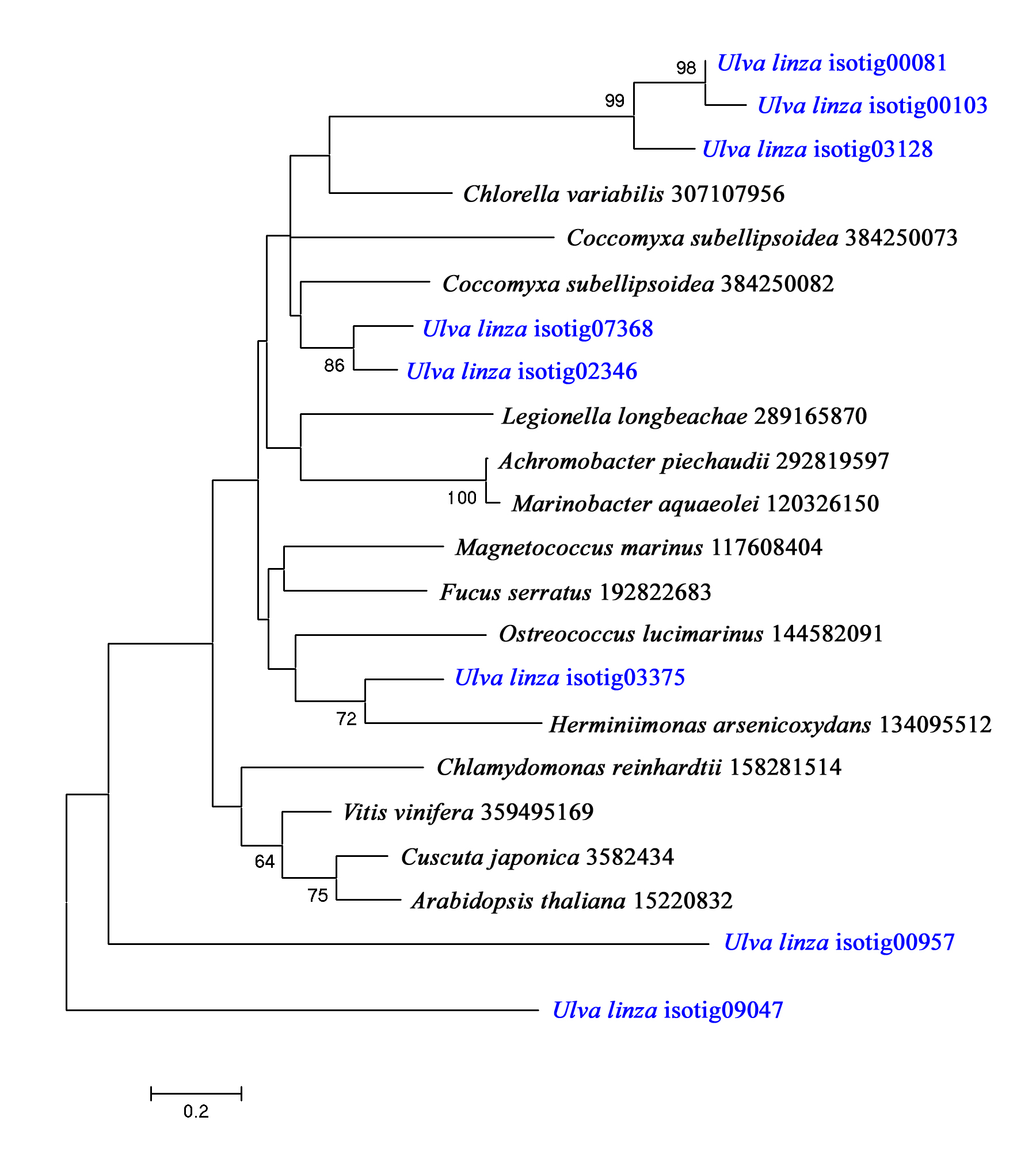

Supplement: Additional file 14 — Figure S9. Phylogenetic analysis of putative Hsp20 proteins in U. linza. The phylogenetic tree was constructed by the neighbor-joining algorithm of the MEGA 4.0 program. A total of 1,000 bootstrap replicates were performed. [file 1471-2164-13-565-S14.jpeg]
