# Supplementary material for: De novo sequencing and analysis of the Ulva linza transcriptome to discover putative mechanisms associated with its successful colonization of coastal ecosystems
Source: BMC Genomics. 2012 Oct 25;13:565. doi: 10.1186/1471-2164-13-565 (PMC3532339; doi:10.1186/1471-2164-13-565)
Supplement: Additional file 5 — Table S4. Putative genes encoding the enzymes required for a C4-like CCM. [file 1471-2164-13-565-S5.doc]

**Additional file 5. Table S4** Putative genes encoding the enzymes required for a C4-like CCM.

| Name | Putative function | E value | Best blast |
| --- | --- | --- | --- |
| isotig01576 | pyruvate phosphate dikinase | 0.0 | *Volvox carteri* EFJ43008.1 |
| isotig05954 | pyruvate phosphate dikinase | 6e-141 | *Ricinus communis* EEF45441.1 |
| isotig03954 | Phosphoenolpyruvate carboxylase | 1e-160 | *Chlamydomonas reinhardtii*  AAS01721.1 |
| isotig03863 | Phosphoenolpyruvate carboxylase | 0 | *Chlamydomonas reinhardtii*  [AAS01722.1](http://www.ncbi.nlm.nih.gov/protein/41387682?report=genbank&log$=protalign&blast_rank=1&RID=0W2UVM8S012) |
| isotig07811 | phosphoenolpyruvate carboxykinase | 4e-162 | *Volvox carteri* EFJ39262.1 |
| isotig03643 | aspartate aminotransferase | 8e-93 | Micromonas sp. RCC299  ACO64018.1 |
| isotig05665 | malate dehydrogenase | 1e-101 | *Volvox carteri* EFJ44027.1 |
| isotig07153 | malate dehydrogenase | 8e-143 | *Chlamydomonas reinhardtii*  EDP03144.1 |
| isotig02937 | NADP-Malate dehydrogenase | 4e-167 | *Chlamydomonas reinhardtii*  EFN59784.1 |
| isotig10629 | NADP malic enzyme | 2e-55 | Coccomyxa subellipsoidea  [EIE27596.1](http://www.ncbi.nlm.nih.gov/protein/384254122?report=genbank&log$=protalign&blast_rank=1&RID=0W518XDJ012) |
