# Supplementary material for: De novo sequencing and analysis of the Ulva linza transcriptome to discover putative mechanisms associated with its successful colonization of coastal ecosystems
Source: BMC Genomics. 2012 Oct 25;13:565. doi: 10.1186/1471-2164-13-565 (PMC3532339; doi:10.1186/1471-2164-13-565)
Supplement: Additional file 13 — Table S5. Putative Heat-shock proteins found in U. linza. Three genes encoding Hsp70, Hsp90 and Hsp100 that not conserved in green algae and plants were shown in yellow color. [file 1471-2164-13-565-S13.doc]

**Additional file 13 Table S5** Putative Heat-shock proteins in *U. linza*.

|  | Name | Putative function | E value | Best blast |
| --- | --- | --- | --- | --- |
| 1 | isotig07368 | Hsp20 | 2e-19 | Magnetococcus marinus ABK43859.1 |
| 2 | isotig02346 | Hsp20 | 3e-19 | Marinobacter aquaeolei ABM20465.1 |
| 3 | isotig03375 | Hsp20 | 2e-18 | Herminiimonas arsenicoxydans CAL62465 |
| 4 | isotig00103 | Hsp20 | 8e-11 | Coccomyxa subellipsoidea EIE23553.1 |
| 5 | isotig00957 | Hsp20 | 2e-10 | Coccomyxa subellipsoidea [EIE23562.1](http://www.ncbi.nlm.nih.gov/protein/384250082?report=genbank&log$=protalign&blast_rank=2&RID=124JAH7A01N) |
| 6 | isotig00081 | Hsp20 | 4e-10 | Coccomyxa subellipsoidea EIE23553.1 |
| 7 | isotig09047 | Hsp20 | 3e-07 | Fucus serratus [ACF06187.1](http://www.ncbi.nlm.nih.gov/protein/192822683?report=genbank&log$=protalign&blast_rank=1&RID=124531XT01S) |
| 8 | isotig03128 | Hsp20 | 1e-04 | Mucilaginibacter paludis [EHQ30217.1](http://www.ncbi.nlm.nih.gov/protein/373894320?report=genbank&log$=protalign&blast_rank=1&RID=1248FGMF01S) |
| 9 | isotig09407 | Hsp20 | 3e-14 | Coccomyxa subellipsoidea EIE23553.1 |
| 10 | contig06648 | Hsp33 | 4e-11 | Chlamydomonas reinhardtii EDO98135.1 |
| 11 | isotig00306 | Hsp70 | 0 | Chlorella variabilis EFN55945.1 |
| 12 | isotig02115 | Hsp70 | 0 | Volvox carteri [EFJ51170.1](http://www.ncbi.nlm.nih.gov/protein/300266985?report=genbank&log$=protalign&blast_rank=2&RID=11VGTJP301S) |
| 13 | isotig04259 | Hsp70 | 0 | Volvox carteri [EFJ49538.1](http://www.ncbi.nlm.nih.gov/protein/300265346?report=genbank&log$=protalign&blast_rank=1&RID=11VP50T201S) |
| 14 | contig01400 | Hsp70 | 0 | Spinacia oleracea [AAB88132.1](http://www.ncbi.nlm.nih.gov/protein/2660768?report=genbank&log$=protalign&blast_rank=5&RID=11XE2TTW012) |
| 15 | isotig04514 | Hsp70 | 5e-93 | Perkinsus marinus EER06446.1 |
| 16 | isotig00454 | Hsp70 | 8e-71 | Coccomyxa subellipsoidea EIE18231.1 |
| 17 | isotig09152 | Hsp70 | 2e-53 | Volvox carteri EFJ48180.1 |
| 18 | isotig10117 | Hsp70 | 4e-43 | Volvox carteri EFJ49538.1 |
| 19 | isotig06120 | Hsp70 | 2e-26 | Coccomyxa subellipsoidea EIE18231.1 |
| 20 | contig14453 | Hsp70 | 2e-15 | Chlamydomonas reinhardtii EDP01195.1 |
| 21 | contig10824 | Hsp70 | 6e-08 | Chlamydomonas reinhardtii EDP01195.1 |
| 22 | isotig04020 | Hsp90 | 0 | Oryza sativa BAD53585.1 |
| 23 | isotig00952 | Hsp90 | 0 | Ulva pertusa BAL45645.1 |
| 24 | isotig04228 | Hsp90 | 0 | Chlamydomonas reinhardtii AAU10511.1 |
| 25 | contig10770 | Hsp90 | 4e-92 | Chara braunii [BAK08668.1](http://www.ncbi.nlm.nih.gov/protein/327164297?report=genbank&log$=protalign&blast_rank=3&RID=120UP6J2013) |
| 26 | contig17307 | Hsp90 | 4e-86 | Coccomyxa subellipsoidea EIE18559.1 |
| 27 | contig15768 | Hsp90 | 7e-68 | Tetrahymena bergeri AAR27543.1 |
| 28 | isotig03974 | Hsp100 | 0 | Chlamydomonas reinhardtii EDP06752.1 |
| 29 | isotig01437 | Hsp100 | 3e-153 | Chlamydomonas reinhardtii EDO99306.1 |
| 30 | isotig06061 | Hsp100 | 2e-103 | Volvox carteri EFJ49084.1 |
| 31 | isotig01436 | Hsp100 | 4e-31 | Leishmania infantum [XP_001466653.1](http://www.ncbi.nlm.nih.gov/protein/146093083?report=genbank&log$=protalign&blast_rank=5&RID=121PNAJ601N) |
| 32 | isotig07405 | Hsp100 | 2e-11 | Prochlorococcus marinus ABM69922.1 |
