# Supplementary material for: De novo sequencing and analysis of the Ulva linza transcriptome to discover putative mechanisms associated with its successful colonization of coastal ecosystems
Source: BMC Genomics. 2012 Oct 25;13:565. doi: 10.1186/1471-2164-13-565 (PMC3532339; doi:10.1186/1471-2164-13-565)
Supplement: Additional file 15 — Table S6. Comparison of Heat-shock proteins among Ulva linza, Micromonas sp. RCC299, Ostreococcus tauri, Chlorella variabilis NC64A, Chlamydomonas reinhardtii, Volvox carteri and Arabidopsis thaliana. [file 1471-2164-13-565-S15.doc]

**Additional file 15 Table S6.** Comparison of Heat-shock proteins among *Ulva linza*, *Micromonas* sp. RCC299, *Ostreococcus tauri, Chlorella variabilis* NC64A*, Chlamydomonas reinhardtii, Volvox carteri* and *Arabidopsis thaliana*.

| organism | Hsp20 | Hsp33 | Hsp70 | Hsp90 | Hsp100 | Total |
| --- | --- | --- | --- | --- | --- | --- |
| *Micromonas* sp. RCC299 | 22 | 2 | 11 | 4 | 2 | 41 |
| *Chlamydomonas reinhardtii* | 8 | 1 | 11 | 4 | 3 | 24 |
| *Ostreococcus tauri* | 6 | 2 | 7 | 4 | 4 | 23 |
| *Chlorella variabilis* NC64A | 12 | 1 | 12 | 4 | 3 | 29 |
| *Volvox carteri* | 9 | 0 | 6 | 4 | 2 | 22 |
| *Ulva linza* | 8 | 1 | 11 | 6 | 5 | 32 |
| *Arabidopsis thaliana* | 13 | 0 | 18 | 7 | 8 | 46 |
