# Supplementary material for: De novo sequencing and analysis of the Ulva linza transcriptome to discover putative mechanisms associated with its successful colonization of coastal ecosystems
Source: BMC Genomics. 2012 Oct 25;13:565. doi: 10.1186/1471-2164-13-565 (PMC3532339; doi:10.1186/1471-2164-13-565)
Supplement: Additional file 16 — Table S7. Primers used in the real-time RT-PCR analysis. [file 1471-2164-13-565-S16.doc]

**Additional file 16 Table S7. Primers used in the real-time RT-PCR analysis**

| primers | Sequences (5’-3’) |
| --- | --- |
| LhcSR-F | CCAGCCATTGTTCACTTCCA |
| LhcSR-R | GACACACGGTATGCCTCACAA |
| psbS-F | TGTTCCACTGAGCGAGACTGA |
| psbS-R | TCATCAACAAAATCACCACTACCC |
| 18S-F | TGCCTAGTAAGCGCGAGTCA |
| 18S-R | AAACGATGGGCAGGGAAAC |
